# Interfacial Tension–Temperature–Pressure–Salinity Relationship for the Hydrogen–Brine System under Reservoir Conditions: Integration of Molecular Dynamics and Machine Learning

**DOI:** 10.1021/acs.langmuir.3c01424

**Published:** 2023-08-31

**Authors:** Sina Omrani, Mehdi Ghasemi, Mrityunjay Singh, Saeed Mahmoodpour, Tianhang Zhou, Masoud Babaei, Vahid Niasar

**Affiliations:** † Department of Chemical Engineering, The University of Manchester, Manchester M13 9PL, United Kingdom; ‡ Institute of Applied Geosciences, Geothermal Science and Technology, Technische Universität Darmstadt, 64289 Darmstadt, Germany; § Group of Geothermal Technologies, Technische Universität Munchen, 80333 Munich, Germany; ∥ College of Carbon Neutrality Future Technology, China University of Petroleum (Beijing), 102249 Beijing, China

## Abstract

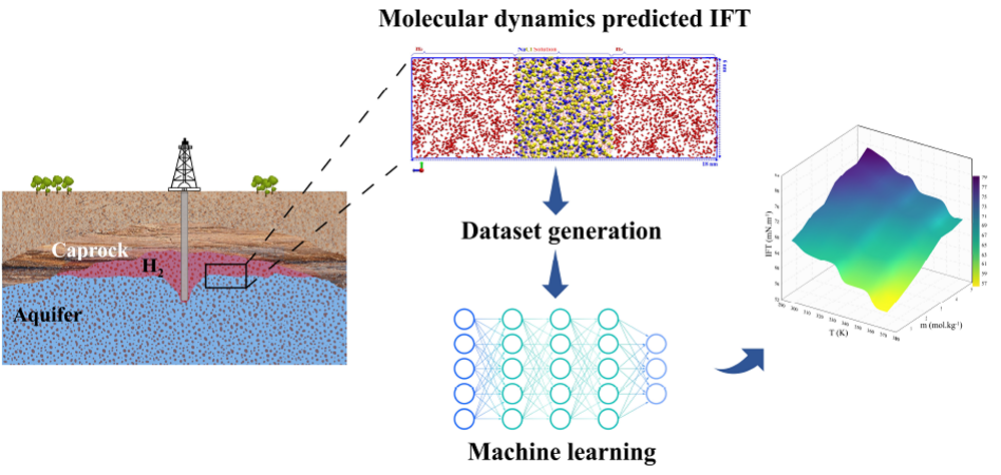

Hydrogen (H_2_) underground storage has attracted considerable attention as a potentially efficient strategy for the large-scale storage of H_2_. Nevertheless, successful execution and long-term storage and withdrawal of H_2_ necessitate a thorough understanding of the physical and chemical properties of H_2_ in contact with the resident fluids. As capillary forces control H_2_ migration and trapping in a subsurface environment, quantifying the interfacial tension (IFT) between H_2_ and the resident fluids in the subsurface is important. In this study, molecular dynamics (MD) simulation was employed to develop a data set for the IFT of H_2_–brine systems under a wide range of thermodynamic conditions (298–373 K temperatures and 1–30 MPa pressures) and NaCl salinities (0–5.02 mol·kg^–1^). For the first time to our knowledge, a comprehensive assessment was carried out to introduce the most accurate force field combination for H_2_–brine systems in predicting interfacial properties with an absolute relative deviation (ARD) of less than 3% compared with the experimental data. In addition, the effect of the cation type was investigated for brines containing NaCl, KCl, CaCl_2_, and MgCl_2_. Our results show that H_2_–brine IFT decreases with increasing temperature under any pressure condition, while higher NaCl salinity increases the IFT. A slight decrease in IFT occurs when the pressure increases. Under the impact of cation type, Ca^2+^ can increase IFT values more than others, i.e., up to 12% with respect to KCl. In the last step, the predicted IFT data set was used to provide a reliable correlation using machine learning (ML). Three white-box ML approaches of the group method of data handling (GMDH), gene expression programming (GEP), and genetic programming (GP) were applied. GP demonstrates the most accurate correlation with a coefficient of determination (*R*^2^) and absolute average relative deviation (AARD) of 0.9783 and 0.9767%, respectively.

## Introduction

The demand for energy is surging, and with the current carbon dioxide (CO_2_) and methane atmospheric emission, the occurrence of an environmental catastrophe originating from global warming is inevitable.^1^ To mitigate CO_2_ emissions, considering a diverse energy portfolio with renewable sources (solar, wind, and electrochemical energy systems) is crucial. However, the dependence of these energy sources on the weather or geological position of the site can lead to a mismatch between seasonal energy demand and supply.^2^ Considering a sustainable energy source, hydrogen (H_2_) has proved to be a low-carbon energy carrier, a cleaner energy source, and a suitable alternative to fossil fuels.^3^ Nevertheless, providing a reliable energy supply and balance in energy demand and supply necessitates an enduring storage of H_2_.^4,5^

Underground salt caverns have been used in the past for the underground storage of H_2_^6,7^ and have higher and cheaper storage capacities compared to those of surface-based H_2_ storage facilities. Although the practicality of H_2_ storage in salt formations has been proved,^8^ a higher storage capacity and sealing capability of aquifers and depleted petroleum reservoirs make them attractive options for large-scale H_2_ storage as well.^9−11^ Nevertheless, successfully storing H_2_ in subsurface geological formations necessitates a comprehensive knowledge of the behavior of H_2_ in the porous storage area under actual thermodynamic conditions.^12^ Two crucial parameters control the H_2_–brine movement in subsurface porous rocks and capillary trapping, which are the interfacial tension (IFT) between H_2_ and water/brine^13^ and the contact angle.^14^ However, the latter results from the interaction between the interfacial tensions at the contact point. In definition, IFT is the key property of the boundary that exists between immiscible fluids and is related to the intermolecular interactions of phases on each side of the boundary.^15^ IFT determines the amount of energy required to spread the boundary^15^ and is considered to be an influential factor in determining the storage capacity, multiphase fluid dynamics, and operational indices.^16^

Considering that the topic is recent, the literature lacks thorough data sets for the IFT of H_2_–water/brine in actual subsurface geological formations. So far, few experimental evaluations have attempted to measure the IFT values (summary in Table 1). Chow et al.^17^ measured the IFT of H_2_–water systems for different temperature and pressure ranges and concluded that an increment in both pressure and temperature leads to a decrease in IFT values. They also provided an empirical correlation for H_2_–water systems covering various thermodynamic conditions (298 < *T* < 448 K and 0.5 < *p* < 45.5 MPa) with an average absolute deviation (AAD) of 0.16 mN·m^–1^ from 129 data points. In addition, Hosseini et al.^18^ considered a brine solution containing NaCl and KCl and noted that increasing salinity increases the IFT of H_2_–brine. The similar effects of pressure and temperature changes on IFT values were also reported for H_2_–brine systems.^18,19^ Al-Mukainah et al.^20^ showed that the IFT of H_2_–brine decreased slightly as the pressure increased. Higgs et al.^21^ measured the IFT of H_2_ in both pure and brine systems and approved that IFT decreased with increasing pressure in pure water while no relationship was observed between IFT and NaCl brine salinities.

**Table 1:** Summary of Interfacial Tension (IFT) Data Experimentally Measured for H_2_-Water/Brine Systems

Reference	Operating Conditions	Salinity Concentration	IFT Range (mN·m^−1^)
Chow et al.^17^	*P* = 0.5 to 45.2 MPa; *T* = 298.03 to 448.35 K	Pure water	42.9 to 73
Yekta et al.^13^	*P* = 5.5 MPa, 10 MPa; *T* = 293.15 K, 313.15 K	Pure water	46 and 51
Higgs et al.^21^	*P* = 0.69 to 20.68 MPa; *T* = 298 K	Pure water; Brine (NaCl): 1000, 2000, 5000 ppm	60.69 to 73.3
Hosseini et al.^18^	*P* = 2.76 to 34.47 MPa; *T* = 298.15 to 423.15 K	Pure water; Brine (NaCl and KCl): 1.05, 3.15, and 4.95 mol·kg^–1^	46.97 to 80.77
Esfandyari et al.^19^	*P* = 1 to 10 MPa; *T* = 293.15 to 353.15 K	Pure water; Formation brine	24.01 to 82.07
Al-Mukainah et al.^20^	*P* = 0.10 to 6.9 MPa; *T* = 323.15 K	Brine (NaCl): 100 000 ppm	51.29 to 63.68

Experimental measurement of the IFT for H_2_ in contact with other fluids can be time- and resource-demanding with high safety risks. An efficient complementary approach to estimating IFT is the molecular dynamics (MD) simulation that can provide accurate information about the interfacial and transport properties of various systems from atomic insight as reported for H_2_ in the literature.^12,22^ Recently, van Rooijen et al.^23^ estimated the IFT properties of the H_2_–NaCl–H_2_O system under various conditions of temperature, pressure, and salinity. They used force fields of TIP4P/2005^24^ for H_2_O, Madrid-2019^25^ and the Madrid-Transport^26^ for NaCl, and the Vrabec^27^ and Marx for H_2_ with about 10% average deviations from experimental data. Doan et al.^28^ predicted H_2_–water IFT values at two different temperatures of 300 and 323 K and pressure ranges of 1–70 MPa. Compared to the experimental data, they underestimated IFT values by about 10–14% due to employing less accurate force fields.

The advent of high-performance computers has facilitated the integration of MD with machine learning (ML) approaches as an alternative to sophisticated mathematical correlation for predicting the desired properties. Recently, coupled MD-ML techniques have been used for forecasting various interfacial properties. For example, Kirch et al.^29^ used MD simulation to create an IFT data set for brine–oil systems under ambient thermodynamical conditions by considering various salinities, brine compositions, oil compositions, and oil density. Then, several ML algorithms, including linear regression (LR), random forest (RF), extra trees (ET), gradient boosted (GB), and elasticNet regression (EN), were implemented to provide a robust model for predicting oil–brine IFT. Zhao et al.^30^ developed a comprehensive database for binary and ternary diffusion coefficients of multicomponent supercritical water (SCW) mixtures using equilibrium MD simulations. The produced data were also used for exploring ML and transfer learning (TL) in predicting the aforementioned types of diffusion coefficients.

### This Study

From the above, it is clear that an extensive study of the IFT prediction for the H_2_–brine system via an accurate force field combination is a missing part. Therefore, a comprehensive evaluation is carried out to assess the accuracy of various force field combinations for the H_2_–brine system. Then, a comprehensive series of MD simulations over a wide range of temperatures (298–373 K), pressures (1–30 MPa), and NaCl salinities (0–5 molality (*m*)), representing the actual conditions of saline aquifers,^31,32^ were conducted. Given that a mixture of salts is present in saline aquifers, the effect of cation type, i.e., K^+^, Ca^2+^, and Mg^2+^, on the IFT and their behavior at the interface of H_2_–brine is scrutinized. Finally, an accurate correlation for IFT prediction with respect to the temperature, pressure, and salinity is presented by integrating the MD, for generating the database, and ML, for training algorithms. This study aims to highlight the importance of these combined techniques as an efficient way to determine the H_2_ properties applicable to subsurface applications. In summary, the following items are the key attributes of this study with respect to the literature:

- Comprehensive accuracy assessment of various force field combinations for the H_2_–brine system to predict the IFT over the actual conditions of saline aquifers.
- Elucidating the effect of cation type, i.e., Na^+^, K^+^, Ca^2+^, and Mg^2+^, on the IFT and their behavior at the interface of H_2_–brine.
- Providing an accurate correlation for IFT prediction with respect to the temperature, pressure, and salinity by integrating MD simulation and ML techniques.

## Methodology

### Molecular Dynamics (MD) Simulation

**Figure 1:**
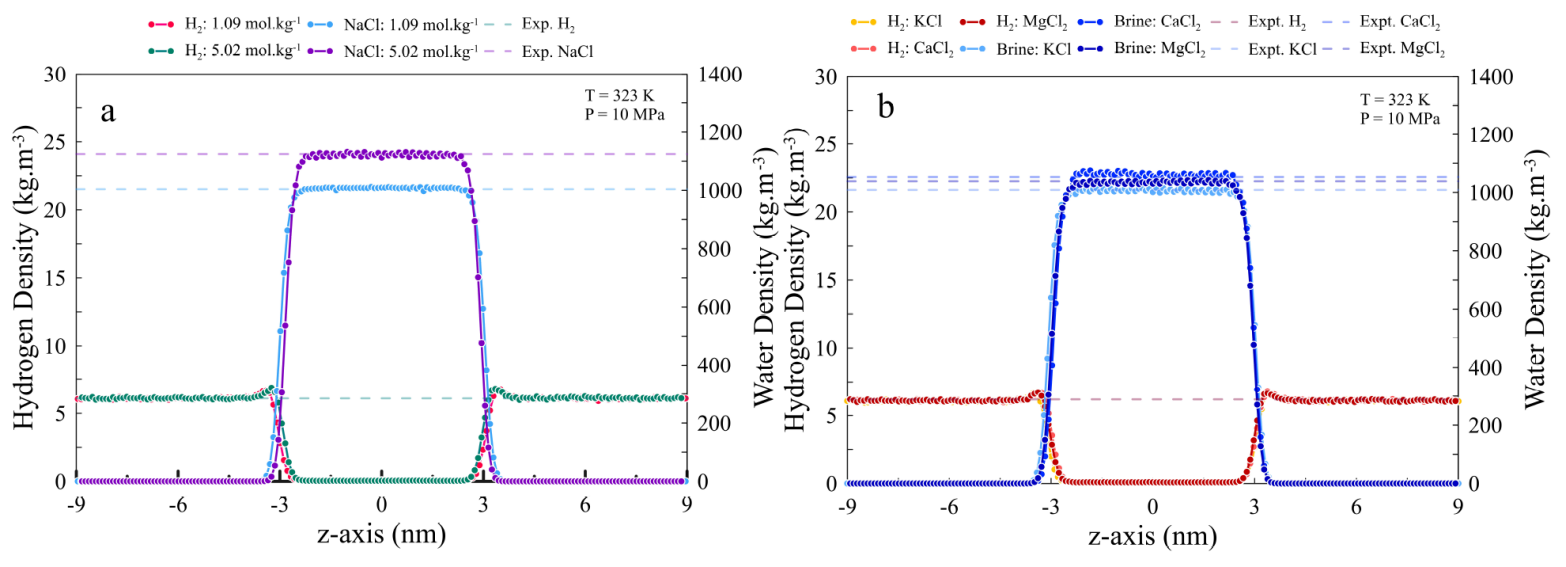
Comparison of the MD-simulated H_2_ and brine densities with the available data in the literature^51,52^ for (a) H_2_–NaCl systems with 1.09 and 5.02 mol·kg^–1^ and (b) H_2_–KCl, H_2_–CaCl_2_, and H_2_–MgCl_2_ systems with a salinity of 1.1 *m*. All comparisons are made at 373 K and 10 MPa. Dashed lines represent experimental values.

All MD simulations were conducted through the GROMACS (version 2021) simulation package.^33^ The accuracy of the MD simulation results significantly depends on the selected atomic force field parameters. In this regard, five H_2_ force fields developed by Vrabec et al.,^27^ Hirschfelder et al.,^34^ Alvai et al.^35^ from modified Silvera–Goldman parameters,^36^ Cracknell,^37^ and Marx and Nielaba^38^ and six potential water models, namely, TIP4P/2005,^39^ modified TIP4P by Rahbari et al.,^40^ TIP4P,^41^ SPC/E,^42^ TIP3P,^41^ and TIP5Pe^43^ that are the most frequent models in the literature to represent H_2_ and water molecules were used to determine the most accurate combined force field parameters to predict H_2_ interfacial properties. The various combinations of the above-mentioned force fields were tested. Their results were compared to two available experimental works on the measurement of IFT for H_2_–water systems conducted by Chow et al.^17^ and Hosseini et al.^18^ Further information about preliminary assessments is provided in the Supporting Information (SI). According to the results and comparisons reported in Table S1, the lowest deviation of IFT predicted by MD simulation from experimental data was obtained by a combination of H_2_ force field parameters developed by Marx and Nielaba^38^ and the TIP4P/2005^39^ water model. Our IFT results showed that the type of water force field plays a critical role in the accuracy of combined force fields. Among all considered cases, only the TIP4P/2005 model could produce IFT data close to experimental values. After validating the accuracy of potential parameters for the H_2_–water system, the most compatible force field parameters for Na^+^ and Cl^–^ ions were also deliberated among those developed by Smith and Dang,^44,45^ Joung and Cheatham^46^ (referred to as SD and JC models), Zeron et al.^25^ (so-called Madrid-2019), and Loche et al.^47^ The results of various combinations for the predicted H_2_–brine IFT, reported in Table S2, showed the smallest difference from experimental data^18^ for the SD force field parameters in conjugation with Marx–TIP4P/2005. Since no experimental data have been reported for the H_2_–brine IFT in the presence of other types of ions, we employed the most frequent force fields available in the literature for predicting the IFT of the gas–brine system.^48,49^ In summary, the Lennard-Jones (LJ) parameters of K^+^ ions were considered from Dang’s work,^45^ and for ions of Ca^2+^ and Mg^2+^, they were obtained from Aqvist’s study.^50^ To prove the accuracy of the selected force fields, the density profiles of H_2_, water, a brine solution of NaCl (1 and 5 *m* concentrations), KCl, CaCl_2_, and MgCl_2_ (1 and 2 *m* concentrations) were precisely compared with the literature. As Figure 1 shows, our predicted density results are in perfect agreement with values reported in the literature^51^ with overall AAD less than 1% from experimental values.

**Figure 2:**
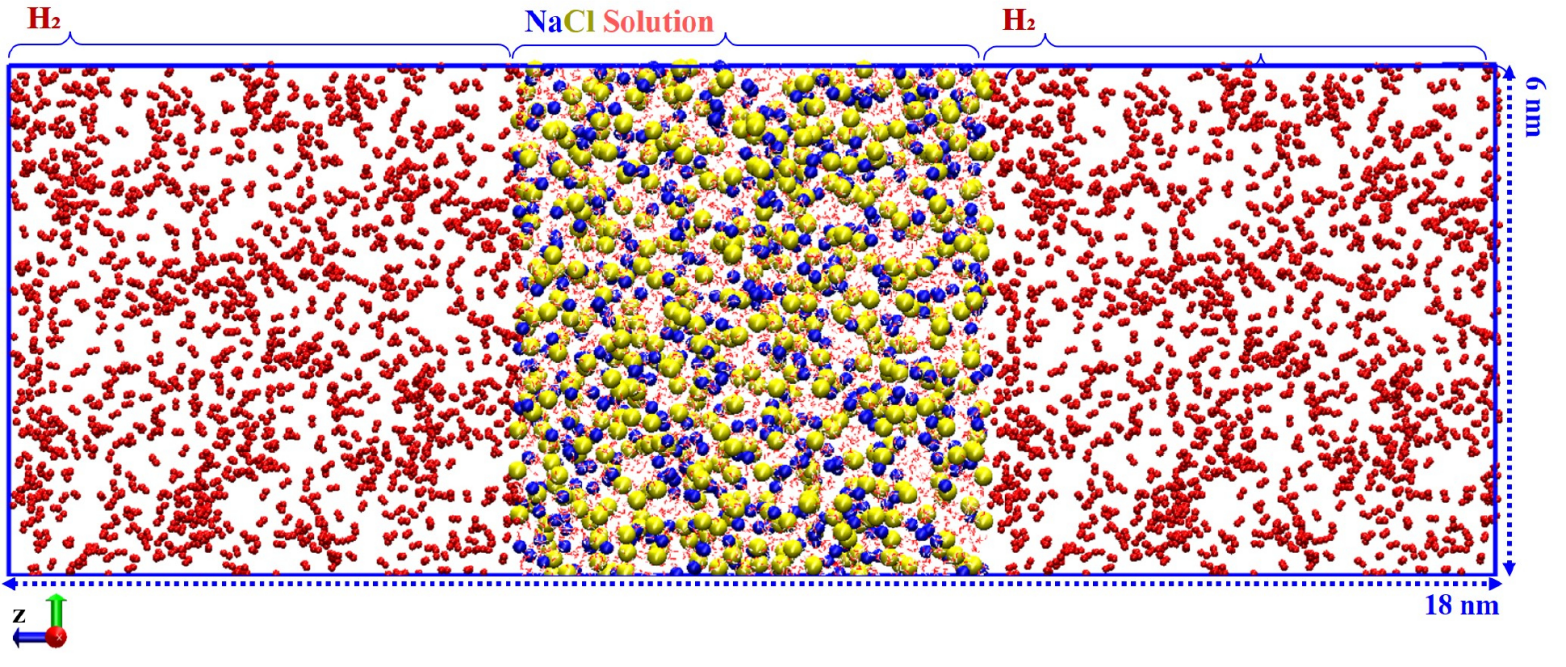
Snapshot of the initial configuration of the simulation system, including NaCl brine solution in the middle of the box surrounded by H_2_ molecules from the two sides with the color coding of red, hydrogen; white, oxygen; blue, sodium; and yellow, chloride.

Figure 2 shows the simulation domain which was generated following the interfacial systems successfully implemented in the literature.^53−57^ First, a water or brine solution box with dimensions of 6 × 6 × 6 nm^3^ was prepared, followed by applying energy minimization of the system using the steepest-descent algorithm to reduce bad contacts among molecules. Next, a 1 ns semi-isotropic *NPT* ensemble was run to bring the system’s density to the desired thermodynamic point. Since the cross-section of the solution box was kept constant, two cubic boxes with dimensions of 6 × 6 × 6 nm^3^ were placed at each end of the water box, as shown in Figure 2. The number of H_2_ molecules was determined based on the density at the desired temperature and pressure.^52^ After the preparation of the initial configuration of the simulation box, the whole system underwent energy minimization again, followed by a 10 ns production run in a canonical (*NVT*) ensemble. IFT values were calculated based on the last nanosecond where the system was totally stabilized. The IFT values were calculated following the Kirkwood–Buff relation^58^ as follows
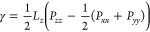
1where γ is the IFT, *L*_*z*_ is the length of the simulation box in the *z* direction and *P*_*xx*_, *P*_*yy*_, and *P*_*zz*_ are the main diagonal values of the pressure tensor. Pressure and temperature were maintained by a Parrinello–Rahman barostat^59^ and a Nosé-Hoover thermostat,^60^ respectively. Periodic boundary conditions (PBCs) were applied in all three directions, and a 2.9 nm cutoff was assigned for nonbonded interactions. Also, for calculating the interatomic potential parameters between unlike atoms, the Lorentz–Berthelot combing rules were employed.^61^ It should be noted that several considerations were taken into account to improve the precision of our model and outcomes. Due to the significant importance of both simulation time and box size in the accuracy of predicted IFT by MD simulation, it is clear that the larger simulation box and the longer simulation time lead to more accurate predictions. However, the computational cost considerably increases. In this regard, further assessments were conducted by evaluating three different simulation times of 3, 5, and 10 ns, four different box sizes, and various cutoff values. As Figure S1 shows, a 10 ns simulation time is long enough to obtain steady IFT results, and that is why the IFT calculation was based on the last nanosecond. According to Table S3, the simulation box with dimensions of 6 × 6 × 18 nm^3^ and with a cutoff value of 2.9 nm, the maximum allowable cutoff, provided the lowest absolute averaged relative deviation in the prediction of IFT compared to the experimental work.^17,18^ Also, all reported results are the average of two repetitions to decrease the statistical uncertainty.

### Machine Learning (ML)

In recent years, with the advent of high-performing computing systems, machine learning (ML) methods have been widely applied in various industries as robust alternative predicative gadgets in order to tackle different aspects of engineering issues.^62^ In this study, the obtained IFT values were considered to be feeds for training ML models to provide an explicit expression for the reliable estimation of the IFT values of H_2_–brine systems as a function of temperature, pressure, and salinity. We employed three white-box ML approaches, including a group method of data handling (GMDH), gene expression programming (GEP), and genetic programming (GP) algorithms. The details of each model can be found in the literature,^63,64^ and a summary of them in addition to the set parameters of each model is presented in the SI. The following structure is acknowledged for the three correlations
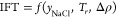
2in which  and *T*_*c*_ = 33.20 *K. y*_NaCl_, *T*_*r*_, *T*_*c*_, and *Δρ* are the NaCl mole fraction, reduced temperature, H_2_ critical temperature, and density difference between H_2_ and brine (kg·m^–3^), respectively. The data set obtained by MD simulation was divided into a training set with 80% of the data set and the remaining 20% used for the testing set. In the development of the GEP and GP correlations, the mean square error (MSE) was considered to be the fitness function to evaluate the chromosomes, which is defined as follows
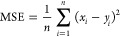
3where *x*_*i*_ and *y*_*i*_ represent the measured and the predicted IFT values.

## Results and Discussion

### Molecular Dynamics Simulation

In this section, we first explain the predicted IFT values and the impacts of the temperature, pressure, and NaCl salinity on them. Then, the impacts of salt type (KCl, CaCl_2_, and MgCl_2_) on the IFT are elaborated. In the final step, a comprehensive comparison is established among the accuracy of employed ML approaches trained by the obtained MD simulation data in predicting IFT.

#### H_2_–NaCl–Water Systems

**Figure 3:**
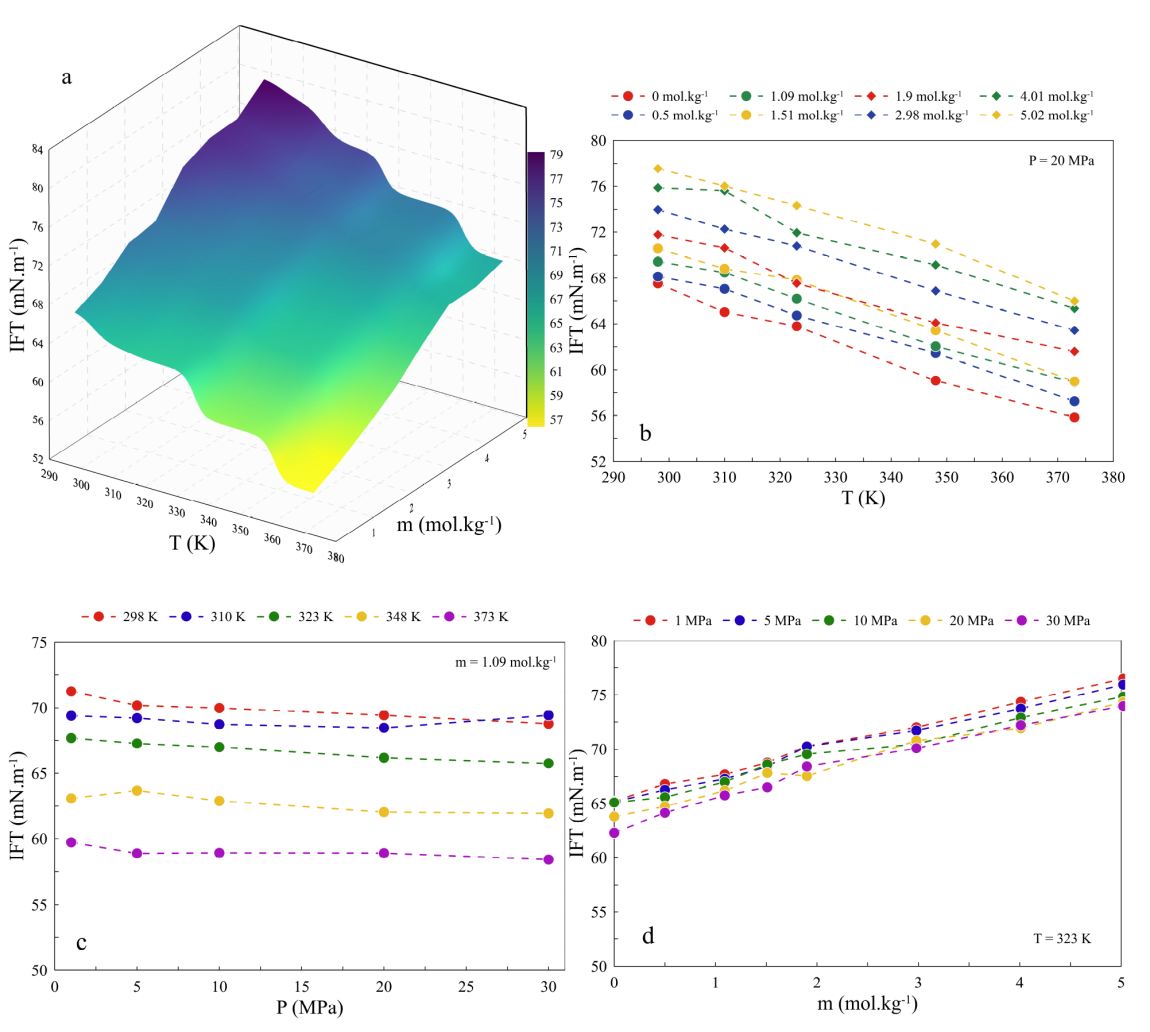
Variation of the predicted IFT values for the H_2_–brine system as a function of (a) temperature (*T* = 298–373 K) and salinity (m = 0–5 mol·kg^–1^) at 10 MPa, (b) temperature and NaCl salinity at 20 MPa, (c) pressure and temperature at 1 *m* NaCl, and (d) pressure and NaCl salinity at 323 K. In the general description, the dependence of IFT on both NaCl salinity and *T* is more significant compared to *P*. An increase in *T* and a decrease in NaCl salinity result in an IFT reduction, while an increase in pressure would not significantly change the IFT.

Figure 3(a) displays the simulated IFT variation for H_2_–NaCl brine systems as a function of the temperature and salinity at the same pressure. Generally, the dependence of IFT on temperature and salinity is analogous to the previous experimental research for H_2_–water/brine,^18,19^ in which at constant pressure and salinity an increase in temperature reduces the IFT and at constant temperature and pressure a higher concentration of NaCl brine leads to an IFT increase. All of the predicted IFT values can be found in Table S6.

In further detail, our results demonstrate that the IFT values are in the range of 55.85 to 77.54 mN·m^−1^ for temperature and NaCl salinity variations of 373 to 298 K and 0 to 5 *m*, respectively, at a constant pressure of 20 MPa. For instance, as Figure 3(b) shows, the predicted IFTs are 67.52 and 55.85 mN·m^−1^ at 298 and 373 K in pure water, a 17% reduction in IFT by increasing the temperature. At the given pressure and NaCl salinity of 5 *m*, IFT decreases by about 15% at 373 K with respect to the IFT of 77.54 mN·m^−1^ at 298 K. Generally, at each pressure, the variation of IFT by temperature increases is between 13 and 18% for salinity ranging from 0 to 5 *m*. The effect of pressure on the IFT of H_2_–brine was also investigated (Figure 3(c)). IFT has no considerable dependence on pressure. Nevertheless, considering all of the cases, the pressure effect can go up to a 6% change in IFT values. At temperatures of 298 and 373 K, the IFT decreases by 3.5% and 2%, respectively, as the pressure increases from 1 to 30 MPa. It is worth mentioning that a slight increase in IFT values with increasing pressure at lower pressures and at some temperatures has also been reported by Chow et al.^17^ for the H_2_–water system. The impact of the NaCl salinity on the IFT is also demonstrated in Figure 3(d). As expected, a greater salinity leads to higher IFT values in all studied cases. Under constant temperature and pressure, a 12–17% increase in IFT occurs as the salinity increases to 5 *m* compared to pure water. Note that at higher temperatures (373 K) the salinity change has a larger impact on the IFT than at lower temperatures (298 K). Also, at the lower salinity of NaCl, the IFT has a more intense response to temperature change than at higher salt concentrations. The IFT values trending with temperature, pressure, and salinity are in agreement with previous experimental work.^17,18^ In comparison to CO_2_ and CH_4_, H_2_–brine IFT has a much lower dependence on the pressure alteration which is due to the lower dependence of the density difference of the H_2_–brine system on pressure.^65^ Generally, increasing the pressure will result in lower IFT values; however, an increase in IFT with increasing pressure at relatively high pressures has been reported for the CH_4_–water system. In addition, an inverse relationship between IFT and salinity or temperature has been reported in both CO_2_– and CH_4_–water systems.^55^

**Figure 4:**
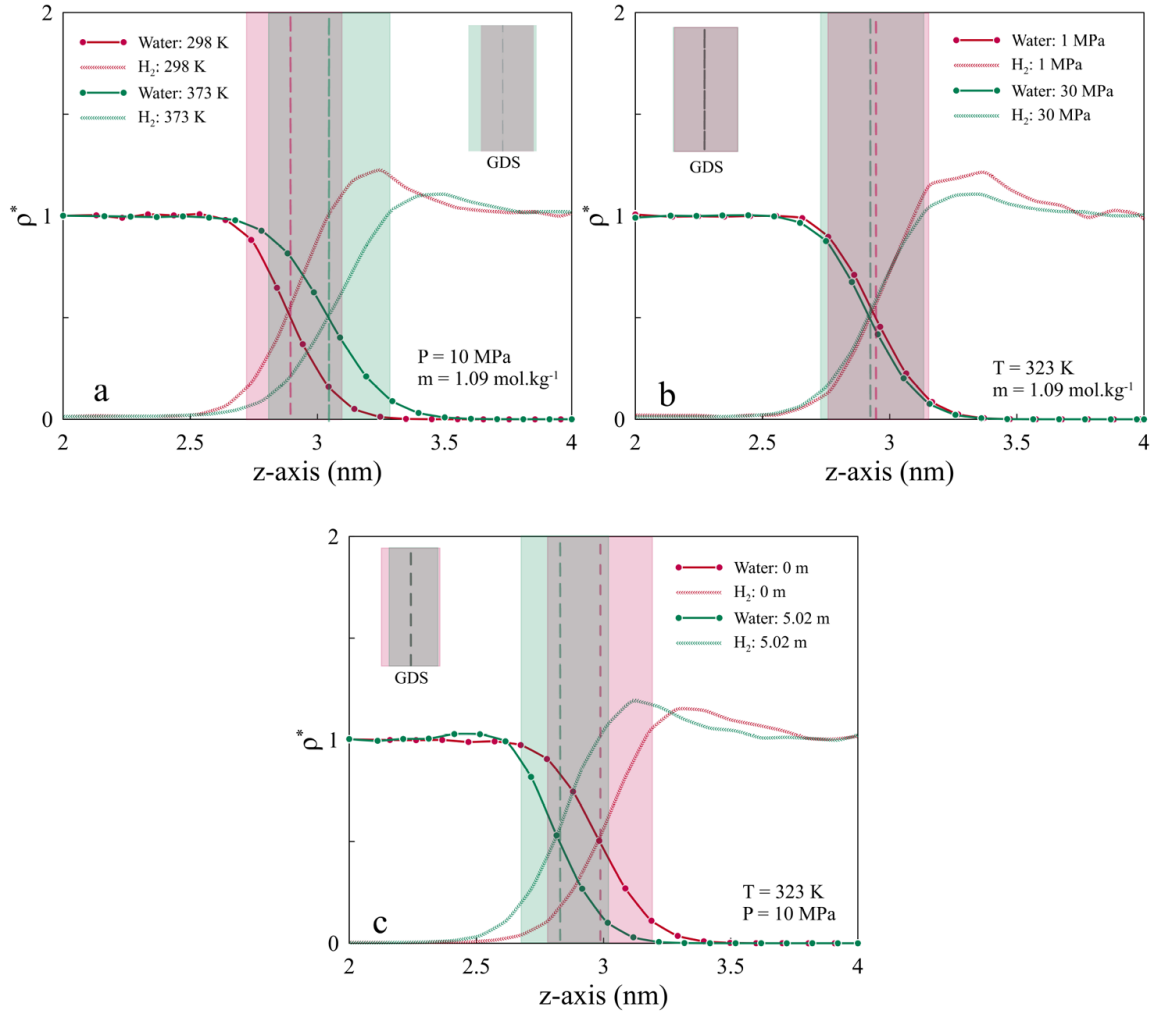
Density profiles of water and H_2_ molecules under the effect of (a) temperature at 1.09 *m* NaCl salinity and 10 MPa pressure, (b) pressure at 1.09 *m* NaCl salinity at 323 K temperature, and (c) salinity at 323 K and 10 MPa pressure. The red and green shadings signify the interfacial width under the mentioned conditions based on the definition of the 10%–90% criterion. For better visualization, first, the center of the brine solution in the *z* direction is considered to be the origin of the figure, and also due to symmetry, only the right-hand side of the simulation box is presented.

Considering the dependence of IFT changes on the atomic structures of the components involved in the interfacial region, molecular concentrations of water, ions, and H_2_ under the effect of temperature, pressure, and salinity are illustrated in Figure 4(a–c). To have a better comparison between the distribution of components, we use the reduced density (ρ*), which is defined as follows^49^
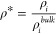
4where ρ_*i*_ is the density of component *i* and ρ _*i*_^*bulk*^ is its bulk density. Since we previously showed that the differences between the density obtained by simulations and references (17) and (18) are less than 1%, the reference for the bulk density of each component is from our simulations. For better visualization in Figure 4, the middle of the brine solution box on the *z* axis is considered to be an origin, and since the simulation box is symmetric, only the right-hand side of the structure is highlighted. Figure 4(a) shows that the effect of temperature on the distributions of water and H_2_ is pronounced, in which the accumulation of H_2_ in the interfacial region decreases as temperature increases.

The Gibbs dividing surface (GDS) was applied to establish the location of the interface. According to the definition, the GDS is placed where the number of excess water molecules on the gas side, i.e., H_2_, equals the deficiency of water molecules on the water side. The distance between the two surfaces at 10% and 90% of the bulk density of the water phase is considered to determine the GDS width.^66^ In Figure 4(a), the red and green shadings represent the interface of H_2_ and water at 298 and 373 K, respectively. As the temperature increases, the GDS width broadens, which means the higher engagement of two phases and, consequently, a lower IFT.

The radial distribution function (RDF), *g*(*r*), curve was used to further demonstrate the engagement by comparing ion pairings between H_2_ and brine compositions, i.e., water, Na^+^, and Cl^–^. According to Figure S5(a), a stronger interaction between H_2_ and water molecules is observed at higher temperatures, indicating a greater possibility of water molecules around the H_2_ interplays since a broadened interfacial width is created. Comparing the left-hand side with the right-hand side of GDS, it is evident that temperature significantly impacts the GDS position and shifts it toward the H_2_ phase. Similarly, an increment in pressure influences the distribution of H_2_ and, to a lesser extent, water molecules. As is evident in Figure 4(b), there is a higher accumulation for H_2_ at 1 MPa than at 30 MPa and faster water depletion at the interface at higher pressure. The thicker interfacial width of H_2_–water at higher pressures indicates the adverse impact of pressure on IFT. The slight variations in intermolecular interactions of H_2_ and water by enhancing pressure, which results in a thicker width, are also approved by RDF peaks, as shown in Figure S5(d). Indeed, there exists a higher RDF peak at higher pressures.

The dependence of composition distributions on NaCl salinity is illustrated in Figure 4(c). When salinity increases, the H_2_ distribution moves toward the brine phase associated with higher accumulation at the interface while water is depleted quickly from the bulk state toward the H_2_ side of the interface. In contrast to the temperature and pressure, less interfacial coverage occurs between H_2_ and water as the NaCl salinity increases, which indicates a direct relationship. In other words, when the enrichment of H_2_ at the interface is significant, a thinner interface width is also observed, implying less mixing of the two phases. Generally, the accumulation of more H_2_ molecules at the interface, either by increasing salinity or decreasing temperature and pressure, leads to milder interactions between H_2_ and water (see Figure S5(a,d,g)), identifying the presence of less water around hydrogen due to thinner interfacial width.

**Figure 5:**
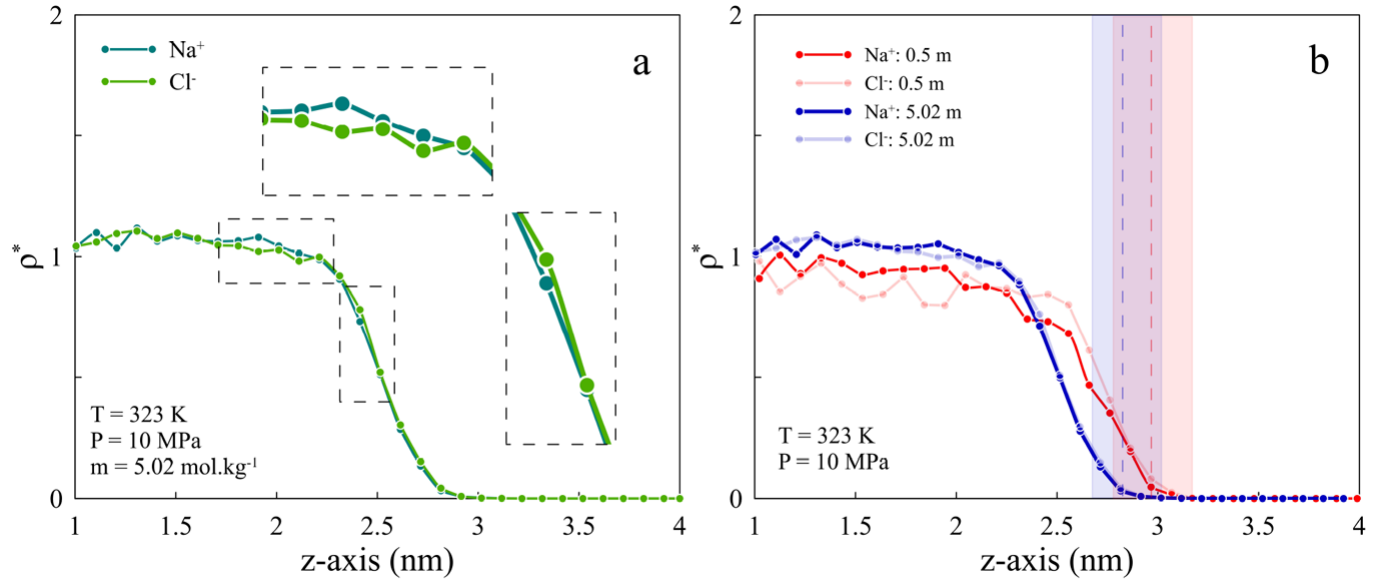
Density profiles of Na^+^ and Cl^–^ ions at the interface of H_2_–water for (a) a salinity of 5.02 *m* to show the tendency of Cl^–^ ions toward the H_2_ phase and (b) salinities of 0.5 and 5.02 *m* to show the tendency of ions’ accumulation in the bulk rather than the interface at higher salinity. This behavior can be examined by comparing radial distribution function (RDF) curves between H_2_ and ions (see Figure S5).

The distribution of ionic compositions in brine is another imperative factor that should be considered for evaluating salinity’s effect on IFT. As is illustrated in Figure 5(a), cations have more tendency toward the bulk phase while a higher depletion rate is observed for anions at the interface. The arrangement of anions at the interface brings about stronger interactions with H_2_ compared to the intensity of interaction between H_2_ and cations, as RDF values show in Figure S5(b,c,e,f,h,i). Similarly, when NaCl salinity increases, ions tend to accumulate in the bulk rather than the interface, as is shown in Figure 5(b). These arrangements of ions influence intermolecular interactions of H_2_ and water, causing a decrease in the possibility of engagement.

#### Effect of Cation Type

**Figure 6:**
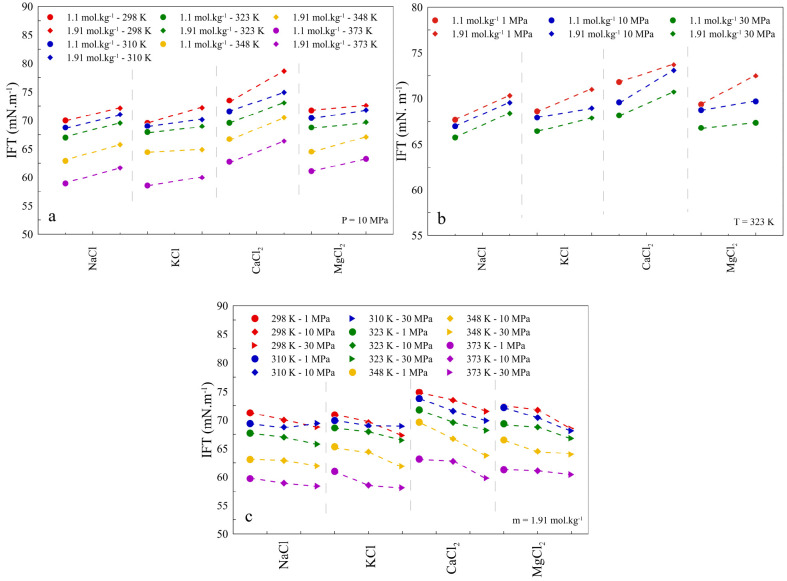
Predicted IFT values between H_2_ and various brine solutions as a function of (a) *T* (K) and salinity (*m*) at *P* = 10 MPa, (b) *P* (MPa) and salinity (*m*) at *T* = 323 K, and (c) *P* (MPa) and *T* (K) at a salinity of 1.91 m.

We previously showed that the higher IFT for H_2_ in the presence of NaCl brine is achieved compared to pure water. Literature highlighted the significant effect of the cation type on CO_2_/CH_4_-brine IFT.^67^ As presented in Figure 6, the impact of the cation type is also pronounced for the IFT of H_2_–brine systems. In the general description, under the same thermodynamic conditions and salinity, a higher IFT is obtained when brine contains CaCl_2_, MgCl_2_, NaCl, and KCl, respectively. For example, under pressure and temperature of 1 MPa and 298 K, respectively, values of 74.87, 72.31, 71.25, and 70.91 mN·m^–1^ were estimated for IFTs of H_2_–brine containing CaCl_2_, MgCl_2_, NaCl, and KCl, respectively, at the identical concentration of 1.1 mol·kg^–1^.

As shown in Figure 6(a), the same as in the NaCl brine case, an increase in temperature brings about a lower IFT for H_2_–brine solutions containing other types of cations. The intensity of the temperature impact on IFT for various brine solution types, namely, CaCl_2_, MgCl_2_, NaCl, and KCl, is the same, in which, under the constant pressure of 10 MPa, the IFT reduction as a result of an increment of temperature from 298 to 373 K is about 15 ± 3% for both salt concentrations, 1.1 and 1.91 mol·kg^–1^. Nevertheless, it can be concluded that the effect of salinity on the IFT is more significant for CaCl_2_. Regarding the impact of pressure, the same as for the NaCl brine, the higher the pressure, the lower the IFT that is achieved for all types of cations. Although the intensity of the pressure impact on the IFT of H_2_–brine containing various cations is different and for the provided case in Figure 6(b), for example, the more considerable the impact that is evident for CaCl_2_ and the minor effect occurs in NaCl brine. This observation cannot be extended to the rest of the conditions, and similar to the two other factors of temperature and salinity, no accurate relationship can be introduced for the intensity of the pressure impact of the IFT vs cation type. All IFT values predicted are listed in Table S7.

**Figure 7:**
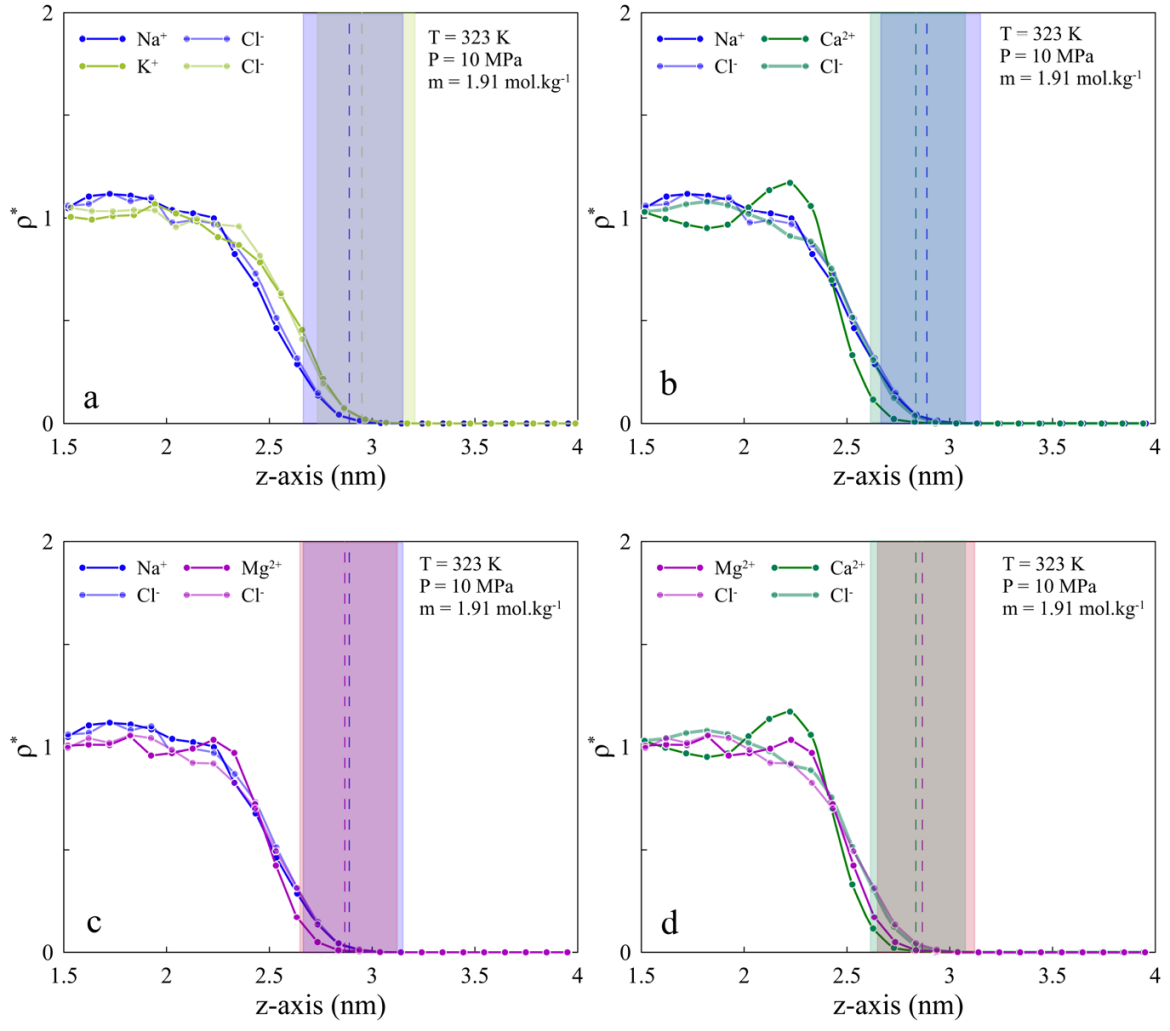
Comparison of ion distribution at the interface of H_2_–brine solution between (a) NaCl and KCl, (b) NaCl and CaCl_2_, (c) NaCl and MgCl_2_, and (d) CaCl_2_ and MgCl_2_ under the same thermodynamic conditions of 323 K, 10 MPa, and 1.91 *m* salinity.

The effect of ion types can be divided into two categories, mono- and divalent cations. It can be discussed from two points of view of how various ion types impact the water configuration and H_2_ distribution at the interface. Although the IFT values are close for both mono- and divalent cations and this lowers the discrepancy between the behavior of the systems, some differences can be detected. Since differences in IFT values become more pronounced at higher salt concentrations, we chose 1.91 *m* for better comparison. Considering the same temperature, pressure, and salinity for all cases (323 K, 10 MPa, and 1.91 *m*), for monovalent cases, KCl shows a different behavior regarding the ion positioning at the interface. As can be seen from Figure 7(a), K^+^ tends to stay on the H_2_ side, and this is confirmed by RDF curves as well (see Figure S7(b and c)). Such behavior for KCl has been reported previously.^68^ Comparing the RDF curves indicates that K^+^ interacts more with H_2_ than does Na^+^, which explains the larger width on the right-hand side of GDS for the KCl system (Figure S6). On the water side, there is a smaller distance between Na^+^–water than the K^+^–water peak, revealing stronger electrostatic interactions between them. In addition, ions in the NaCl system tend to remain in the bulk rather than at the interface compared to KCl, as there is a higher percentage of K^+^ near the GDS (Figure 7(a)). These lead to lower IFT values for the KCl system. In the CaCl_2_ and MgCl_2_ systems, there is a lower interaction between the water system and H_2_ than for the NaCl and KCl cases (see Figure S7). Indeed, the lower engagement for the brine solutions containing divalent cations results in higher IFT values in the H_2_–brine systems. As seen in Figure 7(d), Ca^2+^ has a peak near the interface and accumulates near the interface. Mg^2+^ has weaker but similar behavior. The higher layering structure of divalent ions at the interface has been observed, and their contribution to IFT has also been reported in the literature.^49^ In summary, a comparison among all cases by considering the effects of both salinity and cation types shows that the existence of higher cation molecules at the interface lowers the IFT values. An increase in salinity that results in the presence of a greater number of cations in the bulk compared to the interface causes higher IFT values. For monovalent cations, as K^+^ cations have lower hydration enthalpy (−322 kJ mol^–1^) compared to that of Na^+^ (−406 kJ mol^–1^), they tend to stay at the interface rather than in the bulk. Hence, lower IFT values are obtained for KCl. In addition, the same is true for comparing divalent cations, i.e., Ca^2+^ (−80 kJ mol^–1^) and Mg^2+^ (−74 kJ mol^–1^). The significant difference in the distribution of monovalent and divalent cations at the interface where a higher accumulation of cations observed for the monovalent type is mainly due to cation valency and the layering structure of divalent cations as well as their contributions to IFT.^49^

### Machine Learning

To quantitatively assess the accuracy and performance of GMDH, GEP, and GP algorithms, several statistical parameters including the coefficient of determination (*R*^2^), root-mean-square error (RMSE), absolute average relative deviation (AARD), and graphical assessments such as cross plots of predicted vs measured IFT values, percent relative error distribution, the cumulative frequency diagram, and Williams plot were used.

In the present context, the three statistical indexes are shown for the three ML algorithms in Figure S8. As illustrated, GMDH and GP show very similar accuracy while the GEP algorithm seems to be inferior. As seen in Figure S8(a), the smallest *R*^2^ value is maintained by the GEP algorithm, 0.9672, whereas the corresponding highest value is assumed by the GMDH algorithm, 0.9793, and the GP algorithm has a coefficient of determination of 0.9783. The smallest AARD(%) value, which is more indicative of the accuracy of the predicted IFTs for the GP algorithm, is 0.9767%. However, GDMH has a very close value of 0.9845% and the GEP algorithm shows the highest value of 1.5197%. In addition, the least to greatest RMSE values are for GP, GMDH, and GEP algorithms, with values of 0.6649, 0.6694, and 1.0446, respectively. Overall, the GP algorithm shows better outcomes in predicting the IFT of the H_2_–water–NaCl system.

From the graphical performance measurement perspective, as shown in Figure S9, a good alignment of predicted vs measured data around the slope of unity for GP and GMDH algorithms indicates their better performance, while GEP shows a deviation more notably at lower and higher IFT values. This can be approved by considering the percent relative error graph of the GEP algorithm which is distanced from the zero line at lower and higher IFT values. GP and GMDH show a good distribution around the zero line and consequently more reliability of their correlations. The cumulative frequency diagram in Figure S10 indicates that a higher share of predicted IFT values by GP, GMDH, and GEP algorithms falls into a lower AARD of 1.4%, respectively.

The relevancy factor of input parameters determines which input variables, in our case, density difference (*Δρ*), reduced temperature (*T*_*r*_), and NaCl mole fraction, are most important for predicting the target variable, IFT of H_2_–brine. The relevancy factor is defined as follows
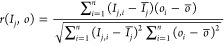
5where *I* and *I̅* are the input parameter and its average, *o* and *o̅* represent the predicted output and its average, and *i* and *j* refer to the data index and the variable, respectively. Therefore, the relevancy factor of the governing input variables is checked on the IFT predictions using the GP algorithm, and it is found that the density difference, reduced temperature, and NaCl mole fraction have the most to least relevancy in IFT values, as shown in Figure S12. In addition, *T*_*r*_ has a negative impact on the IFT values, while *Δρ* and *y*_NaCl_ have a positive impact.

Based on the aforementioned statistical error estimation and model performance assessment, mathematical correlations are developed for each ML algorithm. The GP algorithm showed the best results, and the details of the correlations based on the GMDH and GEP algorithms can be found in the SI. The GP algorithm provides a simple-to-use mathematical expression that can accurately predict the IFT based on the input variables. The developed correlation based on the GP algorithm is shown by eq 6.
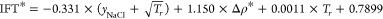
6
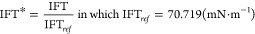
7
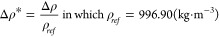
8in which *y*_NaCl_ and *T*_*r*_ are the NaCl mole fraction and reduced temperature, respectively, and IFT* and Δρ* are reduced with respect to a reference point of 298 K, 1 MPa, and 0 mol·kg^–1^. This equation has been validated for the ranges of 298–373 K, 0–5.02 *m*, and 1–30 MPa. Equation 6 was used to predict previous available experimental data by Hosseini et al.^18^ and showed an AARD of 3% over the range covered by our correlation (36 points). This is an acceptable error interval considering that they considered a NaCl–KCl mixture as their brine solution. Recently, van Rooijen et al.^23^ provided a correlation based on MD simulation of the H_2_–water–NaCl system which has an AARD of 7.77% in comparison to the Hosseini et al. experimental work^18^ (considering the same 36 data points). Even though our correlation was developed over the previously mentioned condition, we examined it up to 423 K and 34.47 MPa, which are out of the range of training data. Our correlation obtained an AARD of 2.62% over 12 data points of Hosseini et al.^18^ at 423 K and an AARD of 3.3% over 12 points of their data at 34.47 MPa. This gives a total AARD of 3.11% for all 64 data sets generated by their experimental measurement. These AARD values indicate that our correlation can be used to up to 423 K temperature and 34.47 MPa pressure as well (Table 2). Although the mentioned correlation was developed by only NaCl data, it can be used for other systems of ions provided in this work (up to 1.91 mol·kg^–1^) with 1.65, 2.68, and 2.26% AARD for KCl, CaCl_2_, and MgCl_2_ systems, respectively.

**Table 2:** Absolute Average Relative Error (AARD%) Comparison between Available Correlations for the H_2_–Brine System with Molecular Dynamics (MD) and Hosseini et al.’s Experimental Work^18^

Condition	Number of data points	van Rooijen et al.^23^	This work
298 < *T* < 373	36	7.77	3.00
1 < *P* < 30			
0 < *m* < 5.02			
*T* = 423	12	6.55	2.62
1 < *P* < 30			
0 < *m* < 5.02			
298 < *T* < 373	12	4.37	3.3
*P* = 34.47			
0 < *m* < 5.02			
*T* = 423	4	4.81	4.58
*P* = 34.47			
0 < *m* < 5.02			
298 < *T* < 423	64	6.69	3.11
1 < *P* < 34.47			
0 < *m* < 5.02			

## Conclusions

In this study, MD simulations were conducted to predict the IFT of H_2_–water/brine in a wide range of temperatures (298–373 K), pressures (1–30 MPa), and NaCl salinity (0–5.02 mol·kg^–1^). The effect of cation type on the IFT was also assessed by considering a single salt of KCl, CaCl_2_, and MgCl_2_. In addition, the obtained IFT values were used for training three white-box ML approaches of genetic programming (GP), gene expression programming (GEP), and group method of data handling (GMDH) to provide a robust correlation.

Prior to providing the data set for H_2_–NaCl brine systems and investigating the impacts of the aforementioned factors, we performed an extensive analysis of various combined force fields in predicting IFT using MD simulations. The results of combined force fields were compared with available experimental data in the literature. Among all cases, a combination of Marx-TIP4P/2005-Smith and Dang force fields that describe H_2_, water, and NaCl, respectively, showed the most accurate values against experimental data with a deviation error lower than 3%.

Our findings showed that IFT values had a direct relationship with salinity and an inverse relationship with temperature and pressure. The most and least affecting factors on the IFT of the H_2_–water/brine system are the temperature and pressure, respectively. The impact of the temperature change is more pronounced at lower salinity, while salinity affects IFT values more significantly at higher temperatures. The pressure increment commonly lowers the IFT, although its impact is rather insignificant. This is due to the lower dependence of the density of the system on pressure.

Regarding the cation type effects, CaCl_2_ had the highest IFT values, while KCl had the least influence in most of the cases. The impact of cations is generally a function of their valency and their configuration at the interface. A higher presence of cations at the interface rather than in the bulk lowers the IFT, and each affecting factor, i.e., temperature, pressure, or salinity, changes the IFT through this. Additionally, the GP method showed the best performance in predicting the IFT values with *R*^2^ and AARD% of 0.9783 and 0.9767%, respectively. In summary, MD simulation is a valuable tool not only for providing atomic insight into the interfacial phenomena but also for generating a reliable database that can be considered to be a feed for training ML algorithms in order to expand the database.

## Supporting Information Available

Validation of combined force field models; simulation time and box size; machine learning methodology; IFT results; and machine learning results (PDF)
