# Supplementary material for: Interfacial Tension–Temperature–Pressure–Salinity Relationship for the Hydrogen–Brine System under Reservoir Conditions: Integration of Molecular Dynamics and Machine Learning
Source: Langmuir. 2023 Aug 31;39(36):12680–91. doi: 10.1021/acs.langmuir.3c01424 (PMC10501201; doi:10.1021/acs.langmuir.3c01424)
Supplement: Supplementary file 1 — la3c01424_si_001.pdf [file la3c01424_si_001.pdf]

Supporting Information "Interfacial Tension-Temperature-Pressure-Salinity Relationship for the Hydrogen-Brine System at Reservoir Conditions; Integration of Molecular Dynamics and Machine Learning"

Sina Omrani,[†,‖] Mehdi Ghasemi,[†,‖] Mrityunjay Singh,[‡] Saeed Mahmoodpour,[¶] Tianhang Zhou,[§] Masoud Babaei,[†] and Vahid Niasar[*,†]

†*Department of Chemical Engineering, The University of Manchester, Manchester M13 9PL, United Kingdom*

‡*Institute of Applied Geosciences, Geothermal Science and Technology, Technische Universität Darmstadt, Darmstadt, Germany*

¶*Group of Geothermal Technologies, Technische Universität Munchen, Munich, Germany*

§*College of Carbon Neutrality Future Technology, China University of Petroleum (Beijing), Beijing, China*

‖*S. Omrani and M. Ghasemi contributed equally to this work.*

E-mail: vahid.niasar@manchester.ac.uk;masoud.babaei@manchester.ac.uk

Validation of combined force field models

In order to get the best and most accurate results, we considered various force fields of H_2, water, and ions. These are as follows: Vrabec,[1] Hirschfelder,[2] modified Silvera-Goldman by Alavi et al.,[3] Cracknell,[4] and Marx[5] for H_2, TIP4P/2005,[6] modified TIP4P by Rahbari et al,[7] TIP4P OPLS/AA force field,[8] SPC/E,[9] TIP3P,[8] and TIP5Pe[10] for water, and for ions we used Smith and Dang,[11,12] Joung and Cheatham[13] (we refer to them as SD and

Table S1: Predicted IFT values for the H_2-water system at different thermodynamic conditions using various combined force fields. Comparison with experimental data was also assessed and *ARD* % values were reported in last two columns.

Force Fields	T (K)	P (MPa)	IFT (mN.m^{-1}) This work	IFT (mN.m^{-1}) Chow et al. (*ARD* %)[a]	IFT (mN.m^{-1}) Hosseini et al.[b] (*ARD* %)
Silvera-Goldman - TIP4P OPLS/AA	323	5	50.539	68.9 (26.65)	69.16 (26.26)
Hirschfelder – TIP4P OPLS/AA	323	5	49.276	68.9 (28.48)	69.16 (28.14)
Hirschfelder – TIP4P OPLS/AA	373	20	40.18	58.7 (31.55)	56.51 (30.08)
Cracknell - TIP4P OPLS/AA	323	5	51.421	68.9 (25.36)	69.16 (25.01)
Cracknell – SPC/E	323	5	53.907	68.9 (21.76)	69.16 (21.39)
Marx – modified TIP4P	373	5	44.251	59.5 (25.63)	58.07 (24.39)
Marx – TIP5Pe	373	20	37.395	58.7 (36.29)	56.51 (34.93)
Marx – TIP3P	373	20	36.049	58.7 (38.59)	56.51 (37.27)
Cracknell - TIP4P/2005	373	5	53.808	59.5 (9.57)	58.07 (8.06)
Hirschfelder – TIP4P/2005	373	5	54.272	59.5 (8.79)	58.07 (7.26)
Vrabec – TIP4P/2005	373	5	54.283	59.5 (8.78)	58.07 (7.25)
Silvera-Goldman - TIP4P/2005	373	5	54.226	59.5 (8.86)	58.07 (7.34)
Marx – TIP4P/2005	298	1	70.919	72.9 (0.272)	-
Marx – TIP4P/2005	323	5	65.155	68.9 (5.43)	69.16 (4.99)
Marx – TIP4P/2005	323	20	63.789	67.2 (5.08)	67.58 (4.63)
Marx – TIP4P/2005	373	5	57.133	59.5 (3.98)	58.07 (2.38)
Marx – TIP4P/2005	373	20	55.857	58.7 (4.84)	56.51 (2.80)
Marx – TIP4P/2005	373	30	55.080	-	55.63 (2.97)

[a] Absolute Relative Deviation;
[b] These values are predicted by correlation;

Table S2: Predicted IFT values for the H_2-brine system at different thermodynamic conditions using various brine force fields. Comparison with experimental data was also assessed and ARD % values were reported in last two columns.

Ion Force Field	T (K)	P (MPa)	Salinity (mol.kg^{-1})	IFT (mN.m^{-1}) This work	IFT (mN.m^{-1}) Hosseini et al. (ARD %)
Smith and Dang	298	2.76	1 (NaCl)	71.984	74.97 (3.98)
Smith and Dang	373	2.76	1 (NaCl)	58.377	59.67 (2.17)
Smith and Dang	373	2.76	3 (NaCl)	63.489	63.52 (0.05)
Smith and Dang	323	34.47	1 (0.864 NaCl + 0.136 KCl)	66.279	66.77 (0.74)
Smith and Dang	323	34.47	5 (0.864 NaCl + 0.136 KCl)	74.400	70.51 (5.52)
Smith and Dang	373	2.76	1 (0.864 NaCl + 0.136 KCl)	60.514	59.67 (1.41)
Smith and Dang	373	34.47	1 (0.864 NaCl + 0.136 KCl)	59.768	57.37 (4.05)
Smith and Dang	373	34.47	5 (0.864 NaCl + 0.136 KCl)	66.829	63.22 (5.71)
Joung and Cheatham	373	2.76	1 (NaCl)	59.12	59.67 (0.92)
Joung and Cheatham	373	2.76	1 (0.864 NaCl + 0.136 KCl)	59.741	59.67 (0.12)
Joung and Cheatham	373	2.76	3 (NaCl)	61.88	63.52 (2.58)
Joung and Cheatham	323	34.47	1 (0.864 NaCl + 0.136 KCl)	64.555	66.77 (3.32)
Joung and Cheatham	323	34.47	5 (0.864 NaCl + 0.136 KCl)	66.825	70.51 (5.23)
Joung and Cheatham	373	34.47	1 (0.864 NaCl + 0.136 KCl)	54.97	57.37 (4.18)
Joung and Cheatham	373	34.47	5 (0.864 NaCl + 0.136 KCl)	60.33	63.22 (4.57)
Madrid-2019	373	2.76	3 (NaCl)	59.522	63.52 (6.29)
Loche	323	34.47	5 (0.864 NaCl + 0.136 KCl)	75.875	70.51 (7.61)
Loche	373	34.47	5 (0.864 NaCl + 0.136 KCl)	66.204	63.22 (4.72)

JC models, respectively), Madrid-2019,[14] and Loche et al[15] force fields. The results of various combinations are brought in **Table S1** and **Table S2** for H_2-water and H_2-brine IFT, respectively. We considered two available experimental studies of Chow et al.[16] and Hosseini et al.[17] for validating our models. As can be seen, Marx-TIP4P/2005 combination shows the lowest deviation from experimental data. Our results determined that water force field is of vital importance, and only TIP4P/2005 model can produce data close to

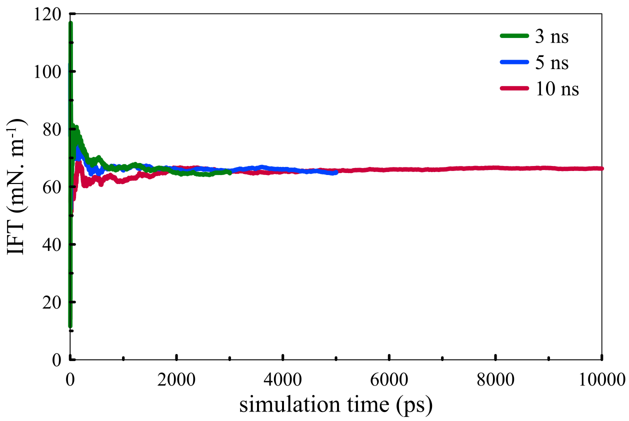

Figure S1: Assessment of the effect of simulation time on variation of IFT. As is evident, the system reaches the stable condition after 2 ns.

experimental values. In addition, various H_2 force fields were examined to have the best possible outcome.

For the H_2-brine system, we considered Marx – TIP4P/2005 with four different ion force fields. The results are reported in **Table S2**. It should be noted that the experimental reference paper used a combination of NaCl and KCl (0.864 NaCl + 0.136 KCl) as their brine mixture. We considered both NaCl and its combination with KCl for validation. Overall, SD ion force field results have the smallest difference from experimental data.

Table S3: Impact of box size with different cut-off values on IFT results.

Simulation box size (nm^3) (x × y × z)	Cut-off (nm)	IFT ($mN.m^{-1}$) This work	IFT ($mN.m^{-1}$) Chow et al. (*ARD* %)	IFT ($mN.m^{-1}$) Hosseini et al. (*ARD* %)
4 × 4 × 12	1.5	54.003	59.5 (9.24)	58.52 (7.72)
4 × 4 × 12	1.9	54.998	59.5 (7.58)	58.52 (6.02)
5 × 5 × 15	1.5	54.248	59.5 (8.83)	58.52 (7.31)
5 × 5 × 15	2.4	55.181	59.5 (7.26)	58.52 (5.71)
6 × 6 × 18	2	57.028	59.5 (4.15)	58.52 (2.56)
6 × 6 × 18	2.9	57.133	59.5 (3.97)	58.52 (2.38)
7 × 7 × 21	3.4	57.008	59.5 (4.18)	58.52 (2.59)

Simulation time and box size

In IFT prediction by MD, simulation time and box size are two vital factors.[18] The larger the simulation box and the longer the simulation time, the more likely it is to achieve more accurate results. However, this condition increases the computational cost significantly. Therefore, it is crucial to find the optimum values of mentioned factors. We considered three different values of 3, 5, and 10 ns for simulation time. The results are presented in **Figure S1**. As seen, 10 ns is long enough to obtain steady IFT results.

As for box size and cut-off distance, various combination was considered, and outcomes are reported in **Table S3**. As the size of the box and cut-off distance increase, the $ARD\%$ decreases and falls into the uncertainty interval of the measured value. It can be deducted that boxes with 6 nm length or higher seem to have a lower and acceptable deviance. The 6×6×18 box with 2.9 nm cut-off distance had the best result with the smallest $ARD\%$.

Machine learning methodology

Group method of data handling (GMDH)

GMDH model is a type of artificial neural network that results in a linear parameter complex polynomial function.[19] The resulting correlation by applying a GMDH model is a polynomial. GMDH composition contains nodes as fundamental components for handling the information. With the presence or absence of an intermediate layer, these nodes are placed in different layers between the input and output layers. The hybrid version of GMDH (HGMDH) admitted the development of the estimation, which was inadequate in the first development by Ivakhnenko in 1971.[20] HGMDH allows interactions among nodes from different layers. This method generates more validity in complex models. The equation of HGMDH is given below:

$$y_i = a + \sum_{i=0}^{m} \sum_{j=1}^{m} ... \sum_{k=1}^{m} \vartheta_{ij...k} x_i^u x_j^u ... x_k^u \qquad u = 1, 2, ..., 2^w \tag{1}$$

Where x and y are the input and the output variables, respectively; $\vartheta_{ij...k}$ stands for the polynomial coefficients; w indicates the number of layers and u is the number of variables. The HGMDH method of computation is described by the following points:

- The below equation determines the correlation of a node N_i with two inputs:

$$N_i^{GMDH} = a_0 + a_1 x_i + a_2 x_j + a_3 x_i x_j + a_4 x_i^2 + a_5 x_j^2 \tag{2}$$

- Computation of polynomial coefficients: the least square procedure is implemented to calculate the resulting coefficients in the expressions of nodes. The following mathematical expression is adjusted:

$$\Delta_j^2 = \sum_{i=1}^{N} (N_i^{GMDH} - y_i)^2 \qquad j = 1, 2, ..., \begin{pmatrix} d \\ 2 \end{pmatrix} \tag{3}$$

where d and N signify the number of variables and data points, respectively.

- Matrix reconstruction: the above equation is modified to a matrix form in order to establish the main mathematical expression:

$$Y = A^T X \tag{4}$$

- The last result is acquired as follows:

$$A^T = y X^T (X X^T)^{-1} \tag{5}$$

The flowchart of the model is shown in **Figure S2**

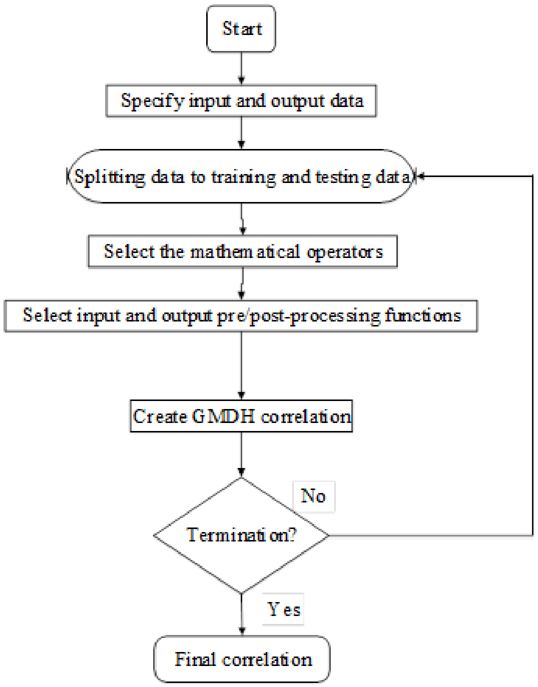

Figure S2: Flowchart of the group method of data handling (GMDH)

Gene expression programming (GEP)

Gene expression programming (GEP) is an evolutionary algorithm that produces computer models and programs. It works based on complicated tree structures by considering the alteration in their sizes, learning, adapting, and shapes.[21] GEP benefits from a genotype–phenotype system, or in another description, from an elementary genome to retain and transfer the genetic information and a complex phenotype to survey the environment and adjust to it. As GEP is in the group of evolutionary algorithms, individuals are selected according to fitness by utilizing populations of individuals and introducing genetic changes using one or more genetic operators.[22] Furthermore, GEP has inherited the linear chromosomes of fixed length from genetic programming that work as the genotype and the parse trees as the phenotype, leading to a genotype/phenotype system.

GEP applies the conventional genetic operators, namely selection, crossover, elitism, and

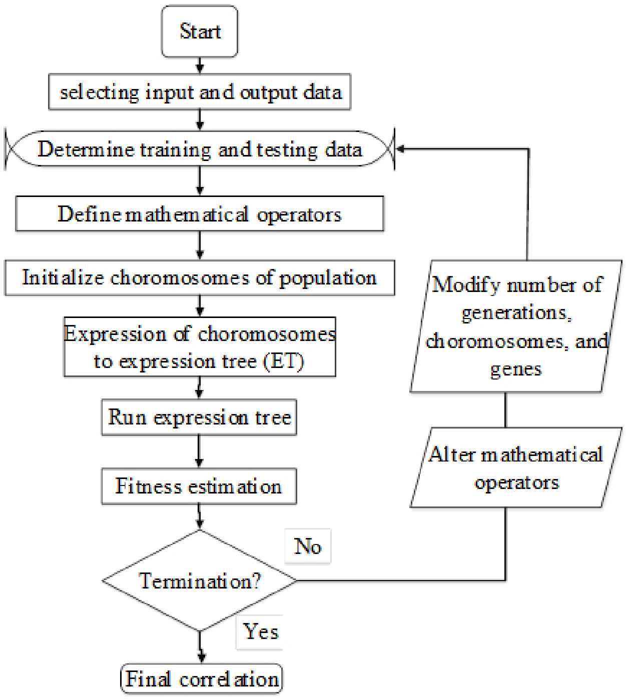

Figure S3: Flowchart of the gene expression programming (GEP)

mutation, and newly executed actions such as transposition and insertion to explore the valid correlations. Besides, the genes have a constant length with terminals that illustrate the variables, such as (x_1, x_2, x_3), and operators such as $+$, $-$, $\times$, $\div$, $\sqrt{}$, ln. The setting parameters are shown in **Table S4**. The flowchart of the model is shown in **Figure S3**.

Table S4: Setting parameters of the gene expression programming (GEN) correlation.

Parameters	Value
Chromosome	100
Gene	2
Operators used	$+$, $-$, $\times$, $/$, $\sqrt{}$, log
Generations	249
Head size	10
Stop condition	Max Fitness
Fitness function	MSE
Used variables	3

Genetic programming (GP)

GP (genetic programming) depends on input and output parameters that determine complicated systems leading to mathematical expressions. This procedure is associated with genetic algorithms which are influenced by the genetic and evolution theories. The common tree-based genetic programming is implemented to govern the individuals (chromosomes), particularly in the iterative operation of GP, which intends to generate the most reliable correlation. The system parameters and the different types of mathematical operators are applied in tree-based genetic programming. The generated chromosomes are evaluated according to a fitness function that mimics the prediction performance of each chromosome. Furthermore, the mean square error function (MSE) is the most common, highly recommended error function. The well-known genetic operators, such as elitism, selection, crossover, and mutation, are employed on the individuals (chromosomes) of the population. Elitism contains selecting a predefined number of chromosomes with the least error and confirming their survival and passage for the succeeding generation. Mutation and crossover are utilized on a group of chromosomes that arise the selection operator to verify the probabilities of mutation and crossover, respectively; thus, they are employed. Different techniques are implemented for the selection, such as the roulette wheel, tournament, and ranking. By securing the probabilities of crossover and mutation, the crossover operator is used to interchange tree segments in chromosomes. In contrast, parts of the chromosomes are altered randomly for the mutation operator. The offspring, known as acquired chromosomes from the genetic operators, configure the new population, which succeeds for the following generation. The mentioned steps are replayed again to the acceptance of the test. The setting parameters are shown in **Table S5**. The flowchart of the model is shown in **Figure S4**

Table S5: Setting parameters of the genetic programming (GP) correlation

Parameters	Value
Population size	30
Operators used	$+, -, \times, /, \surd$
Generations	100
Selection method	Roulette Wheel
Mutation probability	0.30
Crossover probability	0.70
Maximum depth of tree	3
Maximum number of genes per individual	3

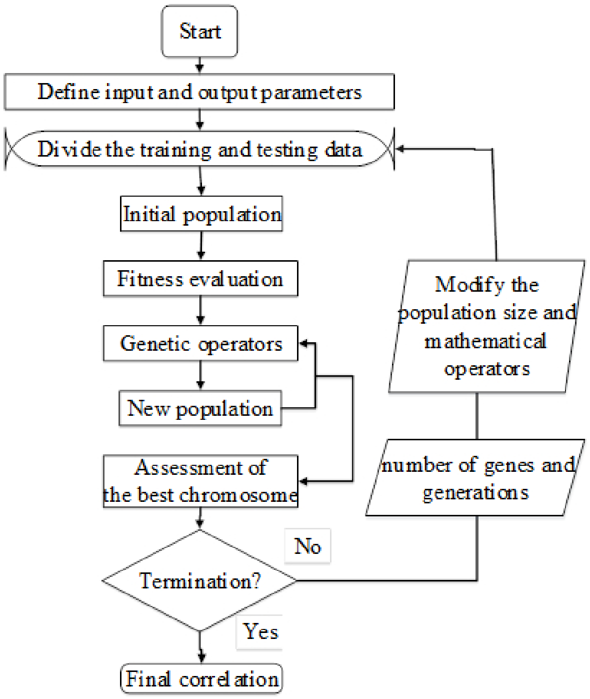

Figure S4: Flowchart of the genetic programming (GP)

IFT results

In the **Table S6**, the IFT values for NaCl salt are reported. We used a naming system to refer to each case, and for simplicity, the values of ion concentration are rounded in naming.

As for other ions considered, **Table S7** present the predicted IFT values in all the considered conditions.

Table S6: IFT values for H_2-water/brine system at various temperatures, pressures, and NaCl salinity.

No	Case Name	T (K)	P (MPa)	Salinity (mol.kg^{-1})	$\Delta\rho$ (kg.m^{-3})	IFT (mN.m^{-1})
1	$T_{298}P_1C_0$	298	1	0	996.90	70.919
2	$T_{298}P_5C_0$	298	5	0	995.03	68.69
3	$T_{298}P_{10}C_0$	298	10	0	993.02	68.233
4	$T_{298}P_{20}C_0$	298	20	0	989.33	67.522
5	$T_{298}P_{30}C_0$	298	30	0	986.02	67.492
6	$T_{310}P_1C_0$	310	1	0	993.56	68.063
7	$T_{310}P_5C_0$	310	5	0	991.84	67.522
8	$T_{310}P_{10}C_0$	310	10	0	989.76	65.459
9	$T_{310}P_{20}C_0$	310	20	0	986.48	65.032
10	$T_{310}P_{30}C_0$	310	30	0	983.34	67.522
11	$T_{323}P_1C_0$	323	1	0	988.62	65.204
12	$T_{323}P_5C_0$	323	5	0	986.97	65.155
13	$T_{323}P_{10}C_0$	323	10	0	985.17	65.096
14	$T_{323}P_{20}C_0$	323	20	0	982.11	63.789
15	$T_{323}P_{30}C_0$	323	30	0	979.12	62.306
16	$T_{348}P_1C_0$	348	1	0	975.46	61.673
17	$T_{348}P_5C_0$	348	5	0	974.08	61.178
18	$T_{348}P_{10}C_0$	348	10	0	972.42	60.693
19	$T_{348}P_{20}C_0$	348	20	0	969.56	59.049
20	$T_{348}P_{30}C_0$	348	30	0	967.06	58.76
21	$T_{373}P_1C_0$	373	1	0	958.36	57.481
22	$T_{373}P_5C_0$	373	5	0	957.39	57.133
23	$T_{373}P_{10}C_0$	373	10	0	955.87	56.378
24	$T_{373}P_{20}C_0$	373	20	0	953.70	55.857
25	$T_{373}P_{30}C_0$	373	30	0	951.45	55.08
26	$T_{298}P_1C_{0.5}$	298	1	0.5	1019.85	71.009
27	$T_{298}P_5C_{0.5}$	298	5	0.5	1017.54	70.158
28	$T_{298}P_{10}C_{0.5}$	298	10	0.5	1015.46	69.405
29	$T_{298}P_{20}C_{0.5}$	298	20	0.5	1011.93	68.124
30	$T_{298}P_{30}C_{0.5}$	298	30	0.5	1008.52	66.813
31	$T_{310}P_1C_{0.5}$	310	1	0.5	1015.82	68.485
32	$T_{310}P_5C_{0.5}$	310	5	0.5	1014.07	68.124
33	$T_{310}P_{10}C_{0.5}$	310	10	0.5	1011.77	67.578
34	$T_{310}P_{20}C_{0.5}$	310	20	0.5	1007.24	67.047
35	$T_{310}P_{30}C_{0.5}$	310	30	0.5	1005.25	65.885
36	$T_{323}P_1C_{0.5}$	323	1	0.5	1010.48	66.802
37	$T_{323}P_5C_{0.5}$	323	5	0.5	1008.60	66.268
38	$T_{323}P_{10}C_{0.5}$	323	10	0.5	1006.58	65.587
39	$T_{323}P_{20}C_{0.5}$	323	20	0.5	1003.28	64.738
40	$T_{323}P_{30}C_{0.5}$	323	30	0.5	1000.25	64.145

Table S6: IFT values for H_2-water/brine system at various temperatures, pressures, and NaCl salinity. (continued)

No	Case Name	T (K)	P (MPa)	Salinity (mol.kg^{-1})	$\Delta\rho$ (kg.m^{-3})	IFT (mN.m^{-1})
41	$T_{348}P_{1}C_{0.5}$	348	1	0.5	996.99	62.633
42	$T_{348}P_{5}C_{0.5}$	348	5	0.5	995.39	62.198
43	$T_{348}P_{10}C_{0.5}$	348	10	0.5	993.74	62.086
44	$T_{348}P_{20}C_{0.5}$	348	20	0.5	990.76	61.464
45	$T_{348}P_{30}C_{0.5}$	348	30	0.5	987.68	60.499
46	$T_{298}P_{1}C_{0.5}$	373	1	0.5	980.09	58.794
47	$T_{298}P_{5}C_{0.5}$	373	5	0.5	979.19	57.275
48	$T_{298}P_{10}C_{0.5}$	373	10	0.5	977.01	57.786
49	$T_{298}P_{20}C_{0.5}$	373	20	0.5	9975.22	57.251
50	$T_{298}P_{30}C_{0.5}$	373	30	0.5	972.66	57.181
51	$T_{298}P_{1}C_{1.09}$	298	1	1.09	1044.38	71.253
52	$T_{298}P_{5}C_{1.09}$	298	5	1.09	1042.59	70.186
53	$T_{298}P_{10}C_{1.09}$	298	10	1.09	1040.27	69.985
54	$T_{298}P_{20}C_{1.09}$	298	20	1.09	1036.17	69.431
55	$T_{298}P_{30}C_{1.09}$	298	30	1.09	1033.39	68.788
56	$T_{310}P_{1}C_{1.09}$	310	1	1.09	1040.07	69.398
57	$T_{310}P_{5}C_{1.09}$	310	5	1.09	1037.90	69.216
58	$T_{310}P_{10}C_{1.09}$	310	10	1.09	1036.12	68.743
59	$T_{310}P_{20}C_{1.09}$	310	20	1.09	1032.20	68.471
60	$T_{310}P_{30}C_{1.09}$	310	30	1.09	1028.35	69.431
61	$T_{323}P_{1}C_{1.09}$	323	1	1.09	1033.69	67.688
62	$T_{323}P_{5}C_{1.09}$	323	5	1.09	1031.92	67.275
63	$T_{323}P_{10}C_{1.09}$	323	10	1.09	1029.71	66.998
64	$T_{323}P_{20}C_{1.09}$	323	20	1.09	1026.31	66.189
65	$T_{323}P_{30}C_{1.09}$	323	30	1.09	1023.43	65.7571
66	$T_{348}P_{1}C_{1.09}$	348	1	1.09	1019.69	63.078
67	$T_{348}P_{5}C_{1.09}$	348	5	1.09	1018.10	63.671
68	$T_{348}P_{10}C_{1.09}$	348	10	1.09	1016.34	62.887
69	$T_{348}P_{20}C_{1.09}$	348	20	1.09	1012.87	62.041
70	$T_{348}P_{30}C_{1.09}$	348	30	1.09	1010.16	61.944
71	$T_{373}P_{1}C_{1.09}$	373	1	1.09	1002.87	59.723
72	$T_{373}P_{5}C_{1.09}$	373	5	1.09	1001.56	58.881
73	$T_{373}P_{10}C_{1.09}$	373	10	1.09	1000.14	58.922
74	$T_{373}P_{20}C_{1.09}$	373	20	1.09	997.13	58.903
75	$T_{373}P_{30}C_{1.09}$	373	30	1.09	994.89	58.409
76	$T_{298}P_{1}C_{1.51}$	298	1	1.51	1060.62	73.371
77	$T_{298}P_{5}C_{1.51}$	298	5	1.51	1058.47	71.65
78	$T_{298}P_{10}C_{1.51}$	298	10	1.51	1056.28	71.72
79	$T_{298}P_{20}C_{1.51}$	298	20	1.51	1052.07	70.582
80	$T_{298}P_{30}C_{1.51}$	298	30	1.51	1048.60	68.945

Table S6: IFT values for H_2-water/brine system at various temperatures, pressures, and NaCl salinity. (continued)

No	Case Name	T (K)	P (MPa)	Salinity (mol.kg^{-1})	$\Delta\rho$ (kg.m^{-3})	IFT (mN.m^{-1})
81	$T_{310}P_{1}C_{1.51}$	310	1	1.51	1055.44	71.054
82	$T_{310}P_{5}C_{1.51}$	310	5	1.51	1053.46	70.582
83	$T_{310}P_{10}C_{1.51}$	310	10	1.51	1051.01	69.862
84	$T_{310}P_{20}C_{1.51}$	310	20	1.51	1047.20	68.808
85	$T_{310}P_{30}C_{1.51}$	310	30	1.51	1044.05	70.582
86	$T_{323}P_{1}C_{1.51}$	323	1	1.51	1048.81	68.758
87	$T_{323}P_{5}C_{1.51}$	323	5	1.51	1047.21	68.408
88	$T_{323}P_{10}C_{1.51}$	323	10	1.51	1044.97	68.585
89	$T_{323}P_{20}C_{1.51}$	323	20	1.51	1041.47	67.819
90	$T_{323}P_{30}C_{1.51}$	323	30	1.51	1038.06	66.502
91	$T_{348}P_{1}C_{1.51}$	348	1	1.51	1034.50	65.418
92	$T_{348}P_{5}C_{1.51}$	348	5	1.51	1032.88	64.723
93	$T_{348}P_{10}C_{1.51}$	348	10	1.51	1031.15	65.081
94	$T_{348}P_{20}C_{1.51}$	348	20	1.51	1027.98	63.439
95	$T_{348}P_{30}C_{1.51}$	348	30	1.51	1025.06	62.758
96	$T_{373}P_{1}C_{1.51}$	373	1	1.51	1017.74	60.847
97	$T_{373}P_{5}C_{1.51}$	373	5	1.51	1016.22	60.654
98	$T_{373}P_{10}C_{1.51}$	373	10	1.51	1014.81	59.683
99	$T_{373}P_{20}C_{1.51}$	373	20	1.51	1012.07	58.985
100	$T_{373}P_{30}C_{1.51}$	373	30	1.51	1009.37	58.56
101	$T_{298}P_{1}C_{1.9}$	298	1	1.9	1078.59	74.663
102	$T_{298}P_{5}C_{1.9}$	298	5	1.9	1076.58	72.791
103	$T_{298}P_{10}C_{1.9}$	298	10	1.9	1073.94	72.127
104	$T_{298}P_{20}C_{1.9}$	298	20	1.9	1070.20	71.768
105	$T_{298}P_{30}C_{1.9}$	298	30	1.9	1066.62	70.512
106	$T_{310}P_{1}C_{1.9}$	310	1	1.9	1073.34	71.796
107	$T_{310}P_{5}C_{1.9}$	310	5	1.9	1071.37	71.768
108	$T_{310}P_{10}C_{1.9}$	310	10	1.9	1068.94	71.023
109	$T_{310}P_{20}C_{1.9}$	310	20	1.9	1065.44	70.621
110	$T_{310}P_{30}C_{1.9}$	310	30	1.9	1061.67	71.768
111	$T_{323}P_{1}C_{1.9}$	323	1	1.9	1066.63	70.288
112	$T_{323}P_{5}C_{1.9}$	323	5	1.9	1064.85	70.261
113	$T_{323}P_{10}C_{1.9}$	323	10	1.9	1062.48	69.537
114	$T_{323}P_{20}C_{1.9}$	323	20	1.9	1059.33	67.535
115	$T_{323}P_{30}C_{1.9}$	323	30	1.9	1055.41	68.402
116	$T_{348}P_{1}C_{1.9}$	348	1	1.9	1051.09	65.473
117	$T_{348}P_{5}C_{1.9}$	348	5	1.9	1049.72	64.964
118	$T_{348}P_{10}C_{1.9}$	348	10	1.9	1047.72	65.721
119	$T_{348}P_{20}C_{1.9}$	348	20	1.9	1044.96	64.092
120	$T_{348}P_{30}C_{1.9}$	348	30	1.9	1042.31	64.068

Table S6: IFT values for H_2-water/brine system at various temperatures, pressures, and NaCl salinity. (continued)

No	Case Name	T (K)	P (MPa)	Salinity (mol.kg^{-1})	$\Delta\rho$ (kg.m^{-3})	IFT (mN.m^{-1})
121	$T_{373}P_1C_{1.9}$	373	1	1.9	1033.82	62.654
122	$T_{373}P_5C_{1.9}$	373	5	1.9	1032.96	61.926
123	$T_{373}P_{10}C_{1.9}$	373	10	1.9	1030.55	61.642
124	$T_{373}P_{20}C_{1.9}$	373	20	1.9	1028.84	61.594
125	$T_{373}P_{30}C_{1.9}$	373	30	1.9	1026.43	60.826
126	$T_{298}P_1C_{2.98}$	298	1	2.98	1109.20	76.097
127	$T_{298}P_5C_{2.98}$	298	5	2.98	1107.65	75.817
128	$T_{298}P_{10}C_{2.98}$	298	10	2.98	1105.16	76.008
129	$T_{298}P_{20}C_{2.98}$	298	20	2.98	1101.50	73.967
130	$T_{298}P_{30}C_{2.98}$	298	30	2.98	1097.02	73.659
131	$T_{310}P_1C_{2.98}$	310	1	2.98	1103.81	73.214
132	$T_{310}P_5C_{2.98}$	310	5	2.98	1101.70	73.967
133	$T_{310}P_{10}C_{2.98}$	310	10	2.98	1099.36	72.934
134	$T_{310}P_{20}C_{2.98}$	310	20	2.98	1095.35	72.259
135	$T_{310}P_{30}C_{2.98}$	310	30	2.98	1091.60	73.967
136	$T_{323}P_1C_{2.98}$	323	1	2.98	1096.30	72.008
137	$T_{323}P_5C_{2.98}$	323	5	2.98	1094.44	71.728
138	$T_{323}P_{10}C_{2.98}$	323	10	2.98	1092.37	70.473
139	$T_{323}P_{20}C_{2.98}$	323	20	2.98	1088.71	70.79
140	$T_{323}P_{30}C_{2.98}$	323	30	2.98	1085.31	70.086
141	$T_{348}P_1C_{2.98}$	348	1	2.98	1080.77	68.239
142	$T_{348}P_5C_{2.98}$	348	5	2.98	1078.86	67.991
143	$T_{348}P_{10}C_{2.98}$	348	10	2.98	1077.14	67.459
144	$T_{348}P_{20}C_{2.98}$	348	20	2.98	1073.89	66.871
145	$T_{348}P_{30}C_{2.98}$	348	30	2.98	1070.81	67.547
146	$T_{373}P_1C_{2.98}$	373	1	2.98	1062.67	64.509
147	$T_{373}P_5C_{2.98}$	373	5	2.98	1062.03	64.487
148	$T_{373}P_{10}C_{2.98}$	373	10	2.98	1060.47	64.338
149	$T_{373}P_{20}C_{2.98}$	373	20	2.98	1056.85	63.429
150	$T_{373}P_{30}C_{2.98}$	373	30	2.98	1054.79	62.787
151	$T_{298}P_1C_{4.01}$	298	1	4.01	1139.57	78.847
152	$T_{298}P_5C_{4.01}$	298	5	4.01	1137.25	77.35
153	$T_{298}P_{10}C_{4.01}$	298	10	4.01	1134.73	77.229
154	$T_{298}P_{20}C_{4.01}$	298	20	4.01	1130.35	75.861
155	$T_{298}P_{30}C_{4.01}$	298	30	4.01	1126.44	75.346
156	$T_{310}P_1C_{4.01}$	310	1	4.01	1132.60	77.153
157	$T_{310}P_5C_{4.01}$	310	5	4.01	1130.94	75.861
158	$T_{310}P_{10}C_{4.01}$	310	10	4.01	1128.32	75.823
159	$T_{310}P_{20}C_{4.01}$	310	20	4.01	1124.53	75.609
160	$T_{310}P_{30}C_{4.01}$	310	30	4.01	1120.52	75.861

Table S6: IFT values for H_2-water/brine system at various temperatures, pressures, and NaCl salinity. (continued)

No	Case Name	T (K)	P (MPa)	Salinity (mol.kg^{-1})	$\Delta\rho$ (kg.m^{-3})	IFT (mN.m^{-1})
161	$T_{323}P_{1}C_{4.01}$	323	1	4.01	1125.37	74.347
162	$T_{323}P_{5}C_{4.01}$	323	5	4.01	1122.51	73.717
163	$T_{323}P_{10}C_{4.01}$	323	10	4.01	1120.86	72.908
164	$T_{323}P_{20}C_{4.01}$	323	20	4.01	1116.98	71.935
165	$T_{323}P_{30}C_{4.01}$	323	30	4.01	1113.37	72.207
166	$T_{348}P_{1}C_{4.01}$	348	1	4.01	1108.82	70.475
167	$T_{348}P_{5}C_{4.01}$	348	5	4.01	1106.96	69.716
168	$T_{348}P_{10}C_{4.01}$	348	10	4.01	1104.38	70.328
169	$T_{348}P_{20}C_{4.01}$	348	20	4.01	1101.70	69.147
170	$T_{348}P_{30}C_{4.01}$	348	30	4.01	1098.49	67.824
171	$T_{373}P_{1}C_{4.01}$	373	1	4.01	1091.09	65.603
172	$T_{373}P_{5}C_{4.01}$	373	5	4.01	1089.44	65.146
173	$T_{373}P_{10}C_{4.01}$	373	10	4.01	1087.84	66.69
174	$T_{373}P_{20}C_{4.01}$	373	20	4.01	1084.71	65.353
175	$T_{373}P_{30}C_{4.01}$	373	30	4.01	1082.01	65.119
176	$T_{298}P_{1}C_{5.02}$	298	1	5.02	1165.50	80.882
177	$T_{298}P_{5}C_{5.02}$	298	5	5.02	1163.59	80.282
178	$T_{298}P_{10}C_{5.02}$	298	10	5.02	1161.14	79.377
179	$T_{298}P_{20}C_{5.02}$	298	20	5.02	1155.92	77.543
180	$T_{298}P_{30}C_{5.02}$	298	30	5.02	1151.79	77.423
181	$T_{310}P_{1}C_{5.02}$	310	1	5.02	1158.52	78.869
182	$T_{310}P_{5}C_{5.02}$	310	5	5.02	1156.42	78.656
183	$T_{310}P_{10}C_{5.02}$	310	10	5.02	1153.76	78.027
184	$T_{310}P_{20}C_{5.02}$	310	20	5.02	1150.03	76.005
185	$T_{310}P_{30}C_{5.02}$	310	30	5.02	1145.33	77.543
186	$T_{323}P_{1}C_{5.02}$	323	1	5.02	1150.30	76.502
187	$T_{323}P_{5}C_{5.02}$	323	5	5.02	1148.30	75.952
188	$T_{323}P_{10}C_{5.02}$	323	10	5.02	1145.76	74.84
189	$T_{323}P_{20}C_{5.02}$	323	20	5.02	1141.52	74.322
190	$T_{323}P_{30}C_{5.02}$	323	30	5.02	1138.31	73.956
191	$T_{348}P_{1}C_{5.02}$	348	1	5.02	1133.55	71.837
192	$T_{348}P_{5}C_{5.02}$	348	5	5.02	1131.66	71.138
193	$T_{348}P_{10}C_{5.02}$	348	10	5.02	1129.69	71.33
194	$T_{348}P_{20}C_{5.02}$	348	20	5.02	1126.02	70.967
195	$T_{348}P_{30}C_{5.02}$	348	30	5.02	1123.12	70.578
196	$T_{373}P_{1}C_{5.02}$	373	1	5.02	1115.49	67.992
197	$T_{373}P_{5}C_{5.02}$	373	5	5.02	1113.04	67.937
198	$T_{373}P_{10}C_{5.02}$	373	10	5.02	1112.53	67.579
199	$T_{373}P_{20}C_{5.02}$	373	20	5.02	1109.27	65.986
200	$T_{373}P_{30}C_{5.02}$	373	30	5.02	1106.37	66.452

Table S7: IFT values for H_2-water/brine system at various temperatures, pressures, and salinity of KCl, $CaCl_2$, and $MgCl_2$.

No	Case Name	T (K)	P (MPa)	Salinity (mol.kg^{-1})	$\Delta\rho$ (kg.m^{-3})	IFT (mN.m^{-1})
201	$T_{298}P_1C_{1.10}S_{KCl}$	298	1	1.10 KCl	1041.96	70.907
202	$T_{298}P_{10}C_{1.10}S_{KCl}$	298	10	1.10 KCl	1038.16	69.549
203	$T_{298}P_{30}C_{1.10}S_{KCl}$	298	30	1.10 KCl	1030.45	67.316
204	$T_{310}P_1C_{1.10}S_{KCl}$	310	1	1.10 KCl	1037.84	69.913
205	$T_{310}P_{10}C_{1.10}S_{KCl}$	310	10	1.10 KCl	1033.71	69.003
206	$T_{310}P_{30}C_{1.10}S_{KCl}$	310	30	1.10 KCl	1026.99	68.93
207	$T_{323}P_1C_{1.10}S_{KCl}$	323	1	1.10 KCl	1045.16	68.623
208	$T_{323}P_{10}C_{1.10}S_{KCl}$	323	10	1.10 KCl	1028.36	67.941
209	$T_{323}P_{30}C_{1.10}S_{KCl}$	323	30	1.10 KCl	1021.91	66.454
210	$T_{348}P_1C_{1.10}S_{KCl}$	348	1	1.10 KCl	1018.79	65.313
211	$T_{348}P_{10}C_{1.10}S_{KCl}$	348	10	1.10 KCl	1015.44	64.406
212	$T_{348}P_{30}C_{1.10}S_{KCl}$	348	30	1.10 KCl	1009.84	61.89
213	$T_{373}P_1C_{1.10}S_{KCl}$	373	1	1.10 KCl	1001.91	60.989
214	$T_{373}P_{10}C_{1.10}S_{KCl}$	373	10	1.10 KCl	999.51	58.562
215	$T_{373}P_{30}C_{1.10}S_{KCl}$	373	30	1.10 KCl	994.96	58.132
216	$T_{298}P_1C_{1.91}S_{KCl}$	298	1	1.91 KCl	1069.63	72.372
217	$T_{298}P_{10}C_{1.91}S_{KCl}$	298	10	1.91 KCl	1065.56	72.198
218	$T_{298}P_{30}C_{1.91}S_{KCl}$	298	30	1.91 KCl	1058.04	69.684
219	$T_{310}P_1C_{1.91}S_{KCl}$	310	1	1.91 KCl	1064.83	70.599
220	$T_{310}P_{10}C_{1.91}S_{KCl}$	310	10	1.91 KCl	1060.79	70.121
221	$T_{310}P_{30}C_{1.91}S_{KCl}$	310	30	1.91 KCl	1053.87	67.256
222	$T_{323}P_1C_{1.91}S_{KCl}$	323	1	1.91 KCl	1058.79	70.972
223	$T_{323}P_{10}C_{1.91}S_{KCl}$	323	10	1.91 KCl	1054.72	68.954
224	$T_{323}P_{30}C_{1.91}S_{KCl}$	323	30	1.91 KCl	1048.18	67.889
225	$T_{348}P_1C_{1.91}S_{KCl}$	348	1	1.91 KCl	1044.83	70.599
226	$T_{348}P_{10}C_{1.91}S_{KCl}$	348	10	1.91 KCl	1042.12	64.86
227	$T_{348}P_{30}C_{1.91}S_{KCl}$	348	30	1.91 KCl	1036.17	66.188
228	$T_{373}P_1C_{1.91}S_{KCl}$	373	1	1.91 KCl	1029.57	61.426
229	$T_{373}P_{10}C_{1.91}S_{KCl}$	373	10	1.91 KCl	1026.55	59.967
230	$T_{373}P_{30}C_{1.91}S_{KCl}$	373	30	1.91 KCl	1021.21	58.995
231	$T_{298}P_1C_{1.10}S_{CaCl_2}$	298	1	1.10 $CaCl_2$	1095.03	74.869
232	$T_{298}P_{10}C_{1.10}S_{CaCl_2}$	298	10	1.10 $CaCl_2$	1090.15	73.479
233	$T_{298}P_{30}C_{1.10}S_{CaCl_2}$	298	30	1.10 $CaCl_2$	1082.52	71.546
234	$T_{310}P_1C_{1.10}S_{CaCl_2}$	310	1	1.10 $CaCl_2$	1090.36	73.777
235	$T_{310}P_{10}C_{1.10}S_{CaCl_2}$	310	10	1.10 $CaCl_2$	1086.08	71.575
236	$T_{310}P_{30}C_{1.10}S_{CaCl_2}$	310	30	1.10 $CaCl_2$	1078.60	69.893
237	$T_{323}P_1C_{1.10}S_{CaCl_2}$	323	1	1.10 $CaCl_2$	1084.54	71.784
238	$T_{323}P_{10}C_{1.10}S_{CaCl_2}$	323	10	1.10 $CaCl_2$	1080.15	69.579
239	$T_{323}P_{30}C_{1.10}S_{CaCl_2}$	323	30	1.10 $CaCl_2$	1072.99	68.173
240	$T_{348}P_1C_{1.10}S_{CaCl_2}$	348	1	1.10 $CaCl_2$	1070.74	69.621

Table S7: IFT values for H_2-water/brine system at various temperatures, pressures, and salinity of KCl, $CaCl_2$, and $MgCl_2$. (continued)

No	Case Name	T (K)	P (MPa)	Salinity (mol.kg^{-1})	$\Delta\rho$ (kg.m^{-3})	IFT (mN.m^{-1})
241	$T_{348}P_{10}C_{1.10}S_{CaCl_2}$	348	10	1.10 $CaCl_2$	1067.40	66.706
242	$T_{348}P_{30}C_{1.10}S_{CaCl_2}$	348	30	1.10 $CaCl_2$	1061.56	63.762
243	$T_{373}P_{1}C_{1.10}S_{CaCl_2}$	373	1	1.10 $CaCl_2$	1055.39	63.143
244	$T_{373}P_{10}C_{1.10}S_{CaCl_2}$	373	10	1.10 $CaCl_2$	1052.43	62.747
245	$T_{373}P_{30}C_{1.10}S_{CaCl_2}$	373	30	1.10 $CaCl_2$	1047.29	59.816
246	$T_{298}P_{1}C_{1.91}S_{CaCl_2}$	298	1	1.91 $CaCl_2$	1151.89	79.121
247	$T_{298}P_{10}C_{1.91}S_{CaCl_2}$	298	10	1.91 $CaCl_2$	1146.99	78.638
248	$T_{298}P_{30}C_{1.91}S_{CaCl_2}$	298	30	1.91 $CaCl_2$	1138.53	75.437
249	$T_{310}P_{1}C_{1.91}S_{CaCl_2}$	310	1	1.91 $CaCl_2$	1147.12	76.635
250	$T_{310}P_{10}C_{1.91}S_{CaCl_2}$	310	10	1.91 $CaCl_2$	1142.06	74.875
251	$T_{310}P_{30}C_{1.91}S_{CaCl_2}$	310	30	1.91 $CaCl_2$	1134.87	73.433
252	$T_{323}P_{1}C_{1.91}S_{CaCl_2}$	323	1	1.91 $CaCl_2$	1140.81	73.685
253	$T_{323}P_{10}C_{1.91}S_{CaCl_2}$	323	10	1.91 $CaCl_2$	1136.28	73.063
254	$T_{323}P_{30}C_{1.91}S_{CaCl_2}$	323	30	1.91 $CaCl_2$	1128.92	70.695
255	$T_{348}P_{1}C_{1.91}S_{CaCl_2}$	348	1	1.91 $CaCl_2$	1127.06	76.635
256	$T_{348}P_{10}C_{1.91}S_{CaCl_2}$	348	10	1.91 $CaCl_2$	1123.49	70.476
257	$T_{348}P_{30}C_{1.91}S_{CaCl_2}$	348	30	1.91 $CaCl_2$	1116.75	69.325
258	$T_{373}P_{1}C_{1.91}S_{CaCl_2}$	373	1	1.91 $CaCl_2$	1110.26	66.184
259	$T_{373}P_{10}C_{1.91}S_{CaCl_2}$	373	10	1.91 $CaCl_2$	1107.69	66.357
260	$T_{373}P_{30}C_{1.91}S_{CaCl_2}$	373	30	1.91 $CaCl_2$	1102.13	65.822
261	$T_{298}P_{1}C_{1.10}S_{MgCl_2}$	298	1	1.10 $MgCl_2$	1063.06	72.314
262	$T_{298}P_{10}C_{1.10}S_{MgCl_2}$	298	10	1.10 $MgCl_2$	1058.56	71.738
263	$T_{298}P_{30}C_{1.10}S_{MgCl_2}$	298	30	1.10 $MgCl_2$	1050.63	68.481
264	$T_{310}P_{1}C_{1.10}S_{MgCl_2}$	310	1	1.10 $MgCl_2$	1059.77	72.19
265	$T_{310}P_{10}C_{1.10}S_{MgCl_2}$	310	10	1.10 $MgCl_2$	1055.44	70.415
266	$T_{310}P_{30}C_{1.10}S_{MgCl_2}$	310	30	1.10 $MgCl_2$	1047.99	68.098
267	$T_{323}P_{1}C_{1.10}S_{MgCl_2}$	323	1	1.10 $MgCl_2$	1054.80	69.364
268	$T_{323}P_{10}C_{1.10}S_{MgCl_2}$	323	10	1.10 $MgCl_2$	1050.24	68.754
269	$T_{323}P_{30}C_{1.10}S_{MgCl_2}$	323	30	1.10 $MgCl_2$	1043.65	66.804
270	$T_{348}P_{1}C_{1.10}S_{MgCl_2}$	348	1	1.10 $MgCl_2$	1043.05	66.498
271	$T_{348}P_{10}C_{1.10}S_{MgCl_2}$	348	10	1.10 $MgCl_2$	1039.93	64.5
272	$T_{348}P_{30}C_{1.10}S_{MgCl_2}$	348	30	1.10 $MgCl_2$	1032.83	63.99
273	$T_{373}P_{1}C_{1.10}S_{MgCl_2}$	373	1	1.10 $MgCl_2$	1029.38	61.272
274	$T_{373}P_{10}C_{1.10}S_{MgCl_2}$	373	10	1.10 $MgCl_2$	1025.94	62.143
275	$T_{373}P_{30}C_{1.10}S_{MgCl_2}$	373	30	1.10 $MgCl_2$	1020.65	60.449
276	$T_{298}P_{1}C_{1.92}S_{MgCl_2}$	298	1	1.92 $MgCl_2$	1102.94	73.521
277	$T_{298}P_{10}C_{1.92}S_{MgCl_2}$	298	10	1.92 $MgCl_2$	1098.39	72.055
278	$T_{298}P_{30}C_{1.92}S_{MgCl_2}$	298	30	1.92 $MgCl_2$	1090.78	71.402
279	$T_{310}P_{1}C_{1.92}S_{MgCl_2}$	310	1	1.92 $MgCl_2$	1098.86	72.931
280	$T_{310}P_{10}C_{1.92}S_{MgCl_2}$	310	10	1.92 $MgCl_2$	1094.73	72.805

Table S7: IFT values for H_2-water/brine system at various temperatures, pressures, and salinity of KCl, $CaCl_2$, and $MgCl_2$. (continued)

No	Case Name	T (K)	P (MPa)	Salinity (mol.kg^{-1})	$\Delta\rho$ (kg.m^{-3})	IFT (mN.m^{-1})
281	$T_{310}P_{30}C_{1.92}S_{MgCl_2}$	310	30	1.92 $MgCl_2$	1087.39	70.129
282	$T_{323}P_{1}C_{1.92}S_{MgCl_2}$	323	1	1.92 $MgCl_2$	1094.42	72.472
283	$T_{323}P_{10}C_{1.92}S_{MgCl_2}$	323	10	1.92 $MgCl_2$	1090.35	69.683
284	$T_{323}P_{30}C_{1.92}S_{MgCl_2}$	323	30	1.92 $MgCl_2$	1083.04	67.366
285	$T_{348}P_{1}C_{1.92}S_{MgCl_2}$	348	1	1.92 $MgCl_2$	1083.53	73.931
286	$T_{348}P_{10}C_{1.92}S_{MgCl_2}$	348	10	1.92 $MgCl_2$	1079.35	67.083
287	$T_{348}P_{30}C_{1.92}S_{MgCl_2}$	348	30	1.92 $MgCl_2$	1072.82	66.057
288	$T_{373}P_{1}C_{1.92}S_{MgCl_2}$	373	1	1.92 $MgCl_2$	1070.24	64.346
289	$T_{373}P_{10}C_{1.92}S_{MgCl_2}$	373	10	1.92 $MgCl_2$	1066.34	63.228
290	$T_{373}P_{30}C_{1.92}S_{MgCl_2}$	373	30	1.92 $MgCl_2$	1060.88	59.622

Machine learning results

Figure S9 shows the percentage error between measured and predicted data for both training and test data set for all three algorithms on the right column, whereas the left column indicates the cross plot of predicted vs. measured values. It is evident that the percentage error is much more minor for GP and GMDH algorithms and spread along the zero line. For the GEP algorithm, the percentage error is skewed for smaller and higher IFTs, and intermediate IFT values, the percentage error is close to zero. This indicates that predictions from the GEP algorithm are better for intermediate IFT values whereas higher accuracy is expected for the GP and GMDH predictor. GMDH is known to generate more complex models than GEP. This means that the GMDH model may be able to capture more of the underlying relationships in the data, leading to better predictions with lower percentage errors. GMDH uses a group-wise optimization technique to select input variables and their interactions, which can result in a more efficient search for the best model. In contrast, GEP uses evolutionary algorithms that may require more computation and be more prone to getting stuck in local optima. The quality and quantity of the input data can also affect the performance of each algorithm. If the GMDH model has access to more or better quality data, it may be able to produce more accurate predictions with lower percentage error. The hyperparameters for each algorithm can also impact their performance.

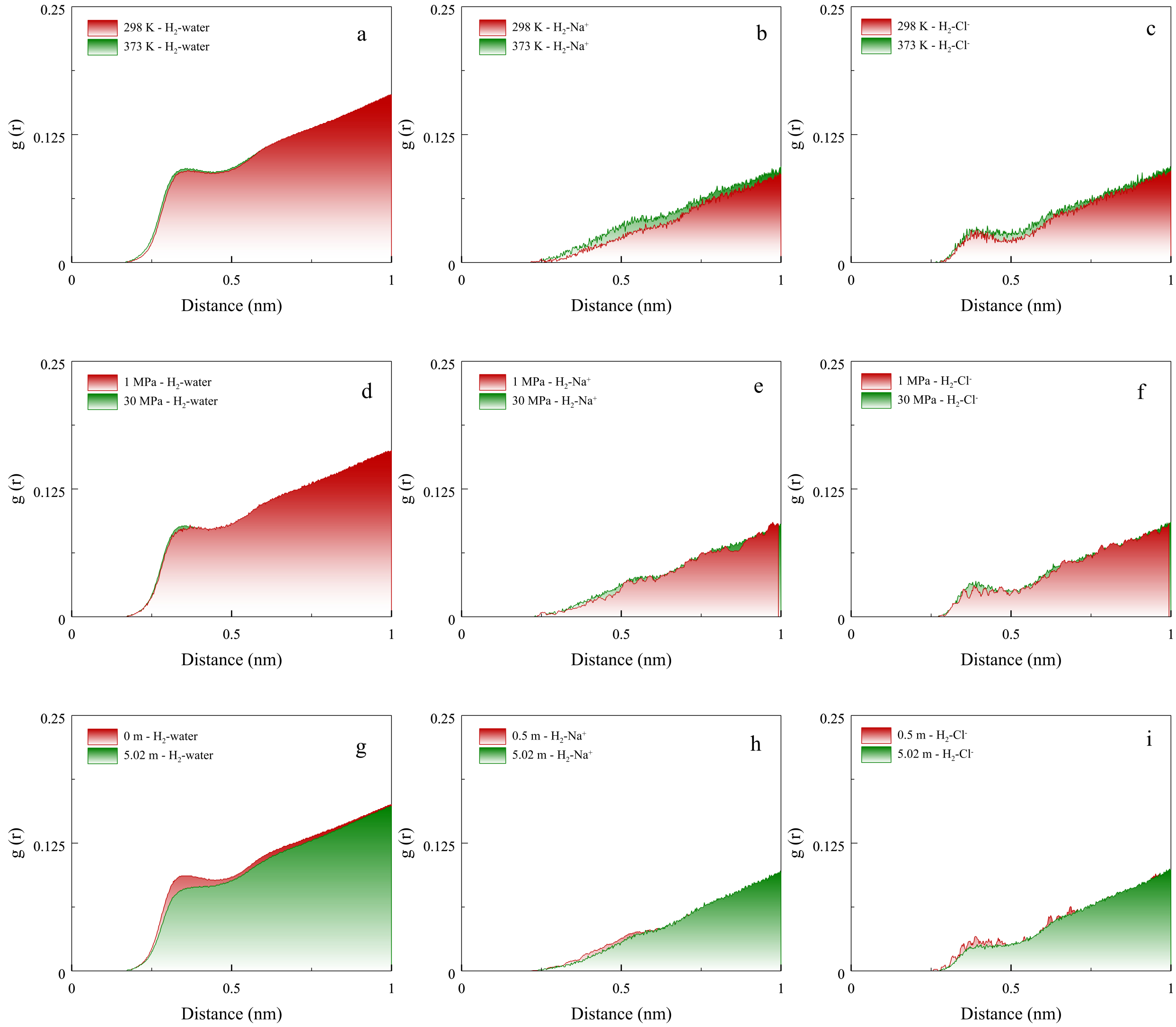

Figure S5: RDF values for a) H_2-water at 298 and 373 K b) H_2-Na^+ at 298 and 373 K c) H_2-Cl^- at 298 and 373 K d) H_2-water at 1 and 30 MPa e) H_2-Na^+ at 1 and 30 MPa f) H_2-Cl^- at 1 and 30 MPa g) H_2-water at 0.5 and 5.02 m h) H_2-Na^+ at 0.5 and 5.02 m i) H_2-Cl^- at 0.5 and 5.02 m

To compare the performance of three algorithms (GMDH, GEP, and GP) in predicting IFT, a cumulative frequency distribution concerning the $AARD$ is determined in **Figure S10**. The $AARD$ is a common performance metric used in statistical modeling, and it measures the absolute difference between the predicted and actual values, normalized by the actual value. The cumulative frequency distribution concerning the $AARD$ shows the percentage of predictions that fall within certain ranges of $AARD$. To construct the cumulative

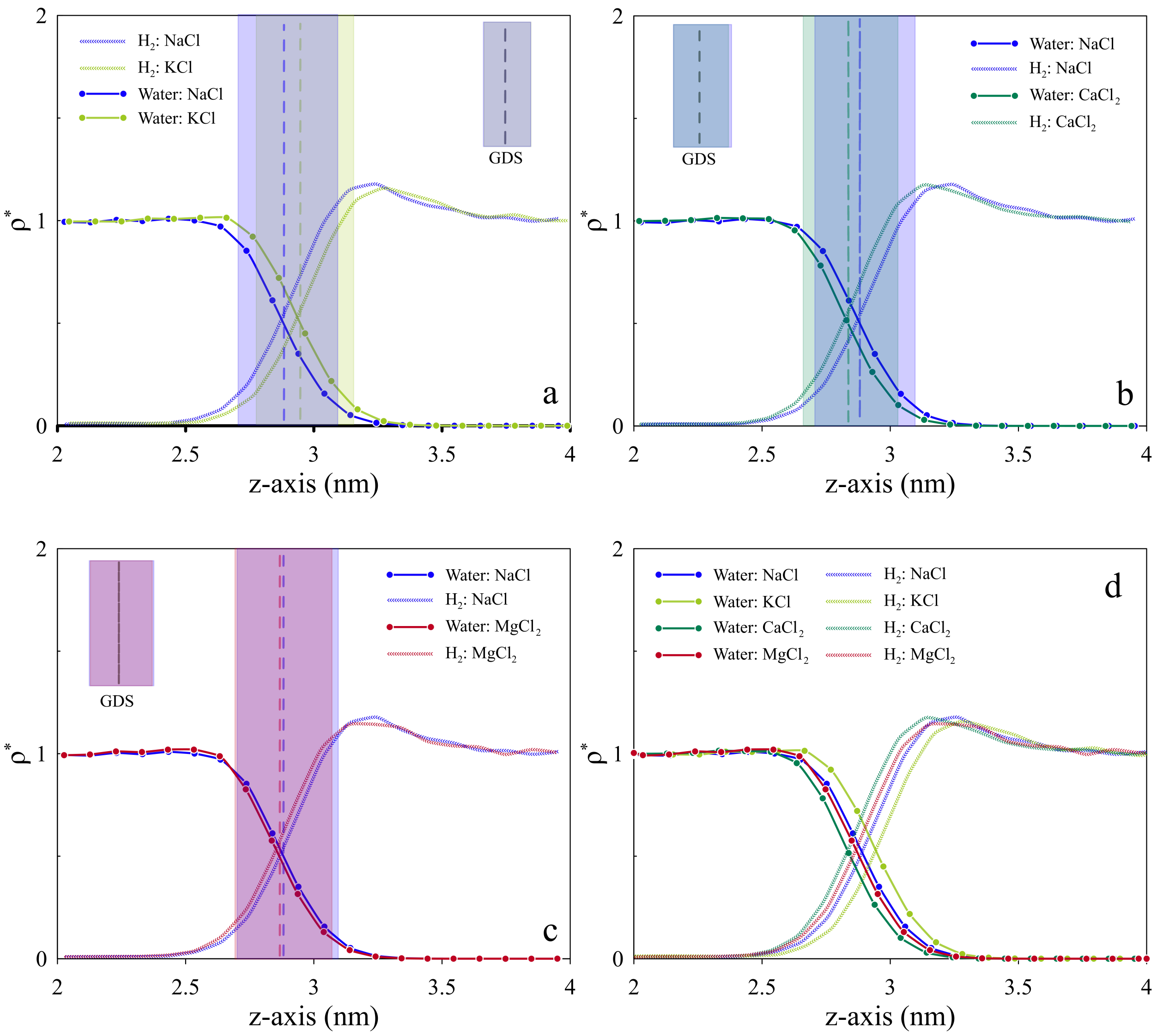

Figure S6: Density of water and H_2 molecules and interfacial width for (a) KCl and NaCl, (b) NaCl and $CaCl_2$, (c) NaCl and $MgCl_2$, and (d) all cases together at the temperature of 323 K, the pressure of 10 MPa, and salinity of 1.91 m

frequency distribution, the *AARD* for each prediction made by each algorithm is calculated in the beginning. Then, the *AARD* values are sorted in ascending order and divided into bins of equal width. Following this, the number of predictions is counted that fall into each bin for each algorithm and therefore, the cumulative frequency for each bin is calculated. An algorithm that has a higher percentage of predictions within a lower *AARD* range is considered to be more accurate. It is evident from **Figure S10** that the cumulative frequency for

GMDH and GP are the bests and very close to each other.

The standardized residuals for the three algorithms, GP, GEP, and GMDH, can be compared using the Williams plot as shown in **Figure S11**. To create the plot, the models are trained on the same dataset, generating predictions on a hold-out dataset. The standardized residuals for each prediction are then calculated by dividing the residual by the standard deviation of the residuals. The Williams plot is examined to compare the accuracy of the models across the range of predicted values. The figure shows that the residuals are randomly distributed around zero, indicating that the models accurately predict the target variable. In contrast, systematic patterns in the residuals could indicate bias or missing features.

The correlation is developed by using the GEP algorithm.

$$IFT = A + B \tag{6}$$

$$A = y_{NaCl} \times \log(T_r^{T_r} \times \Delta\rho \times T_r + y_{NaCl} * T_r - \Delta\rho^2) \tag{7}$$

$$B = T_r + \sqrt{T_r + y_{NaCl}} - \frac{T_r^{y_{NaCl}} - (\Delta\rho + T_r)}{2T_r} \tag{8}$$

Unlike the other two algorithms, which use a fixed set of input variables or a predetermined model structure, the GMDH algorithm generates a set of candidate models with varying levels of complexity by iteratively selecting subsets of input variables and evaluating their performance. The algorithm then combines the best-performing models to create a final, highly complex model that can capture the underlying relationships between the input variables and the output variable with high accuracy. This can result in a highly complex model with many terms that capture the various relationships between the input variables and the output variable as shown by Equations 9 and 10. The complexity of the model can be useful for accurately predicting the IFT, but may also make the model difficult to interpret and understand.

$$IFT = 31.8173 + (1570.38 \times y_{NaCl}^2) - (22.8693 \times A \times y_{NaCl}^2) + 0.00780767 \times A^2 \tag{9}$$

$$A = 607.71 + 3.32623 \times \ln(T_r) \times (\sqrt{\Delta\rho}) - 31.7045 \times \ln T_r^2 - 34.9591 \times \sqrt{\Delta\rho} + 0.487895 \times \Delta\rho \tag{10}$$

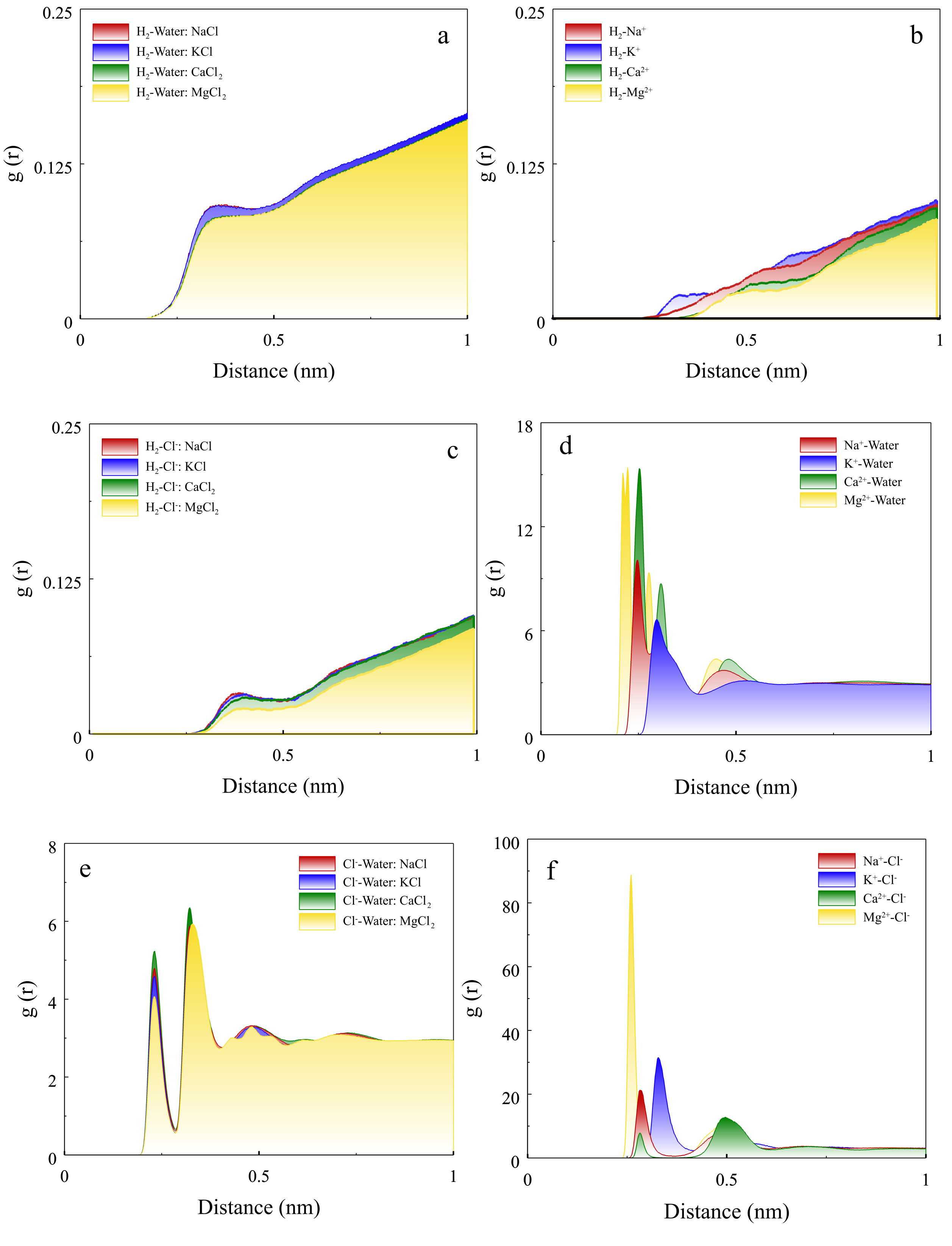

Figure S7: RDF curves for (a) H_2-water, (b) H_2-cation, (c) H_2-Cl^-, (d) cation-water, (e) Cl^--water, and (f) cation-Cl^- for various studied cases containing NaCl, KCl, $CaCl_2$, and $MgCl_2$ at 323 K, 10 MPa, and 1.91 m.

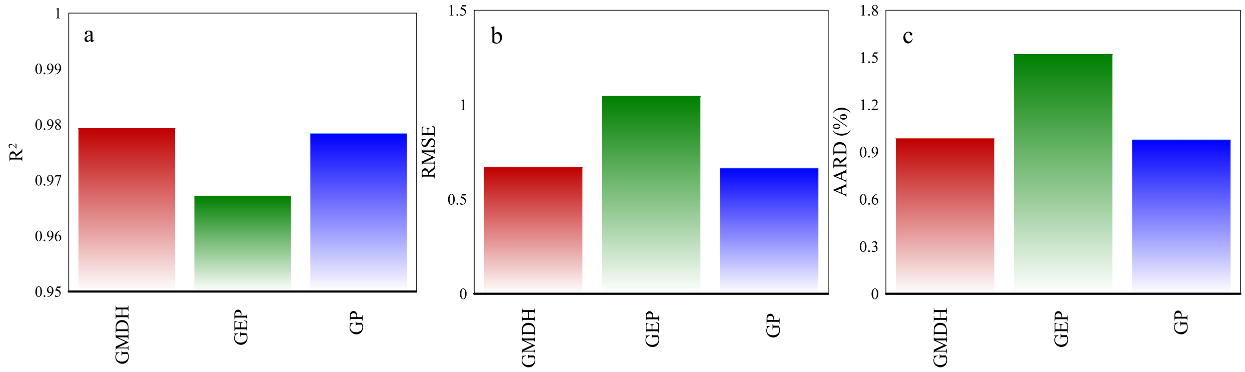

Figure S8: Performance comparison for different proposed models by R^2, $RMSE$, and $AARD\%$

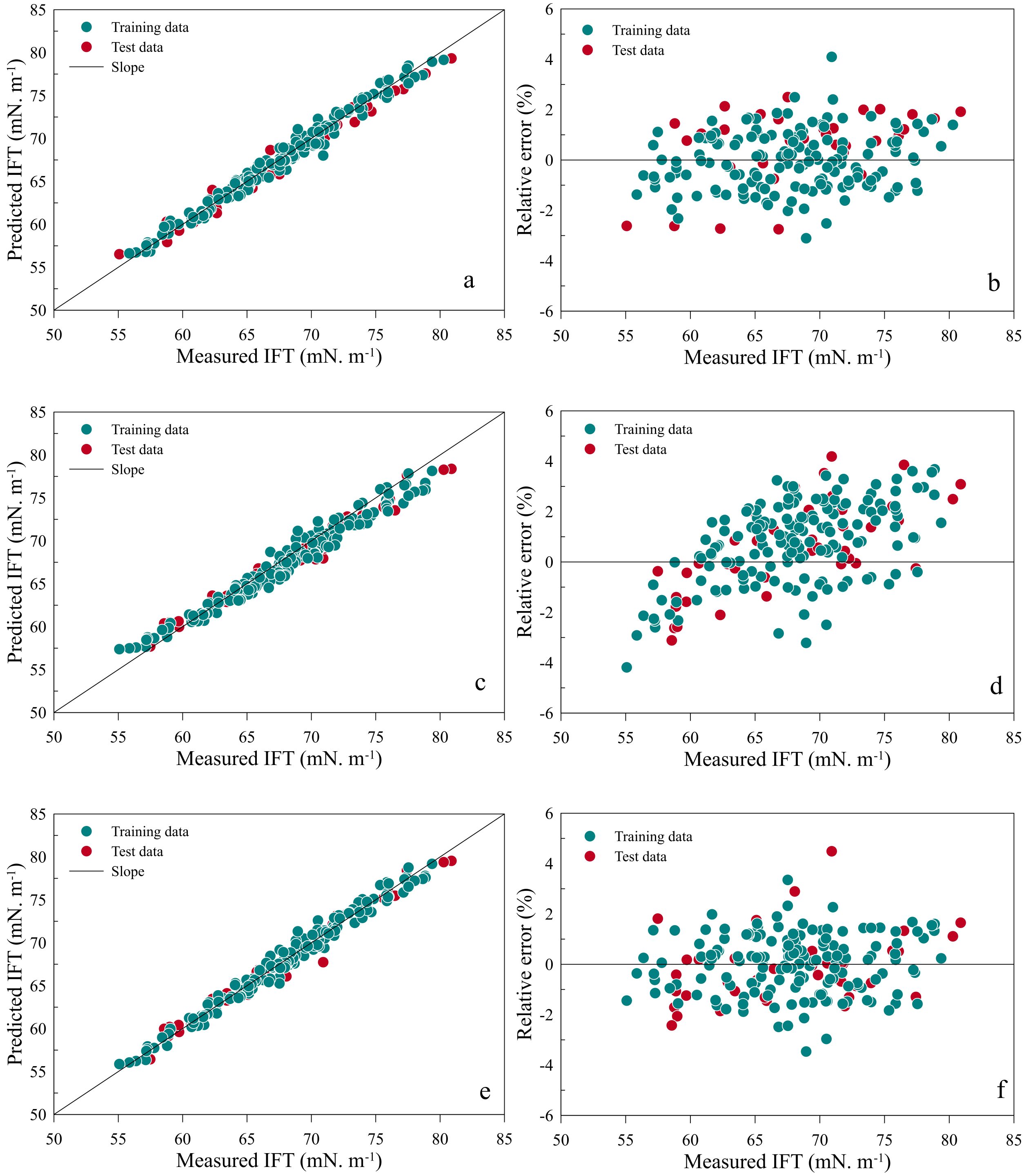

Figure S9: Comparison of IFT and relative error concerning the measured IFT: (a and b) GMDH, (c and d) GEP, and (e and f) GP.

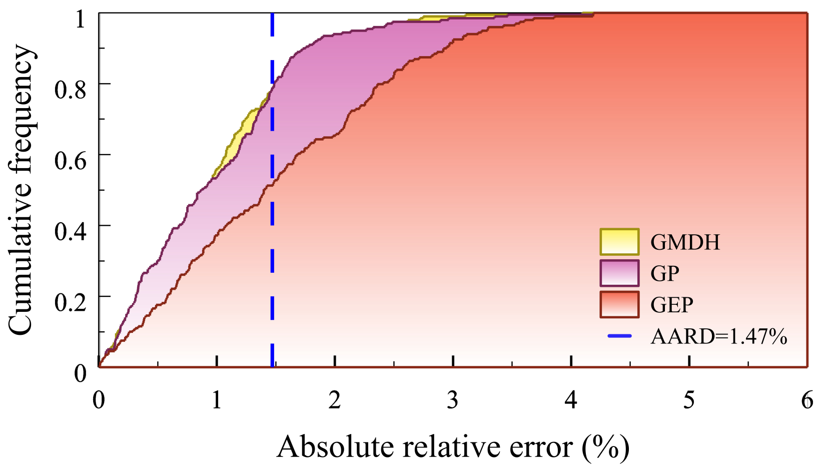

Figure S10: Cumulative frequency concerning absolute relative error for three proposed models.

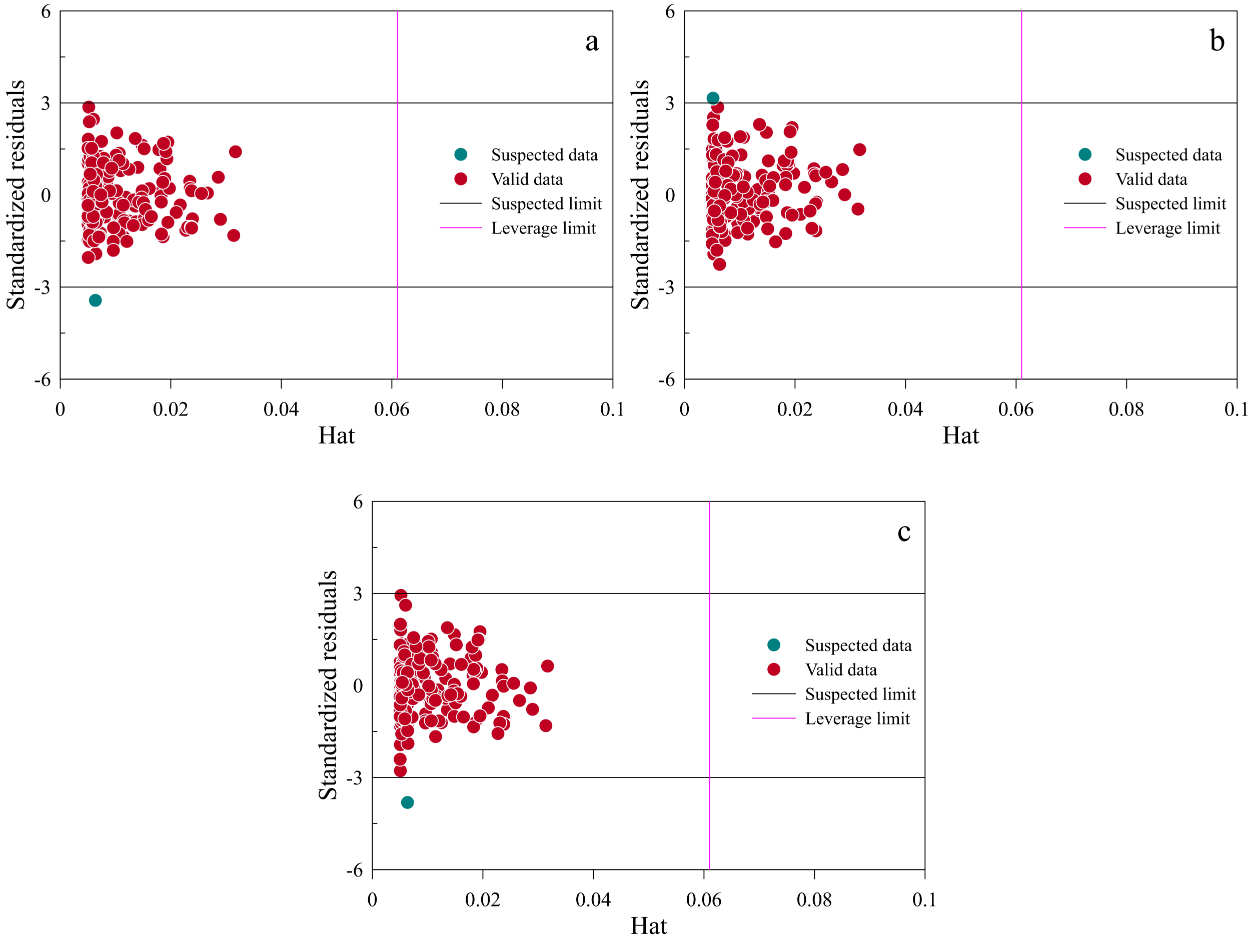

Figure S11: Williams plot to compare three proposed models: (a) GMDH, (b) GEP, and (c) GP.

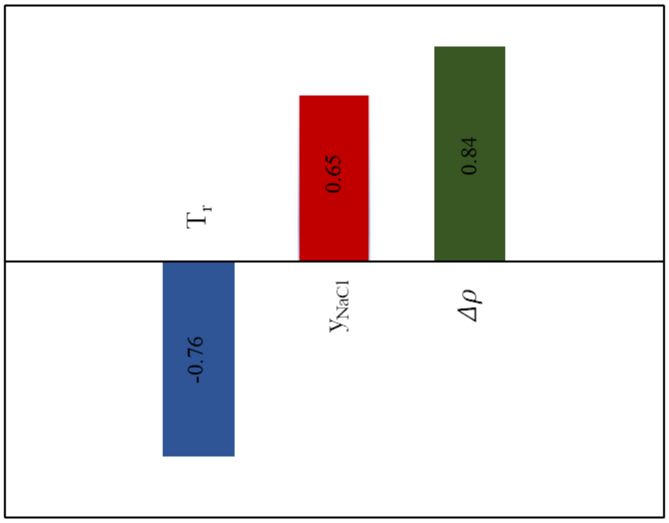

Figure S12: Relevancy factor of input parameters.

References

(1) Koster, A.; Thol, M.; Vrabec, J. Molecular models for the hydrogen age: hydrogen, nitrogen, oxygen, argon, and water. *Journal of Chemical & Engineering Data* **2018**, *63*, 305–320.

(2) Hirschfelder, J. O.; Curtiss, C. F.; Bird, R. B. Molecular theory of gases and liquids. *Molecular theory of gases and liquids* **1964**,

(3) Alavi, S.; Ripmeester, J.; Klug, D. Molecular-dynamics study of structure II hydrogen clathrates. *The Journal of chemical physics* **2005**, *123*, 024507.

(4) Cracknell, R. F. Molecular simulation of hydrogen adsorption in graphitic nanofibres. *Physical Chemistry Chemical Physics* **2001**, *3*, 2091–2097.

(5) Marx, D.; Nielaba, P. Path-integral Monte Carlo techniques for rotational motion in two dimensions: Quenched, annealed, and no-spin quantum-statistical averages. *Physical Review A* **1992**, *45*, 8968.

(6) Abascal, J. L.; Vega, C. A general purpose model for the condensed phases of water: TIP4P/2005. *The Journal of chemical physics* **2005**, *123*, 234505.

(7) Rahbari, A.; Garcia-Navarro, J. C.; Ramdin, M.; Van Den Broeke, L. J.; Moultos, O. A.; Dubbeldam, D.; Vlugt, T. J. Effect of water content on thermodynamic properties of compressed hydrogen. *Journal of Chemical & Engineering Data* **2021**, *66*, 2071–2087.

(8) Jorgensen, W. L.; Chandrasekhar, J.; Madura, J. D.; Impey, R. W.; Klein, M. L. Comparison of simple potential functions for simulating liquid water. *The Journal of chemical physics* **1983**, *79*, 926–935.

(9) Berendsen, H.; Grigera, J.; Straatsma, T. The missing term in effective pair potentials. *Journal of Physical Chemistry* **1987**, *91*, 6269–6271.

(10) Rick, S. W. A reoptimization of the five-site water potential (TIP5P) for use with Ewald sums. *The Journal of chemical physics* **2004**, *120*, 6085–6093.

(11) Smith, D. E.; Dang, L. X. Computer simulations of NaCl association in polarizable water. *The Journal of Chemical Physics* **1994**, *100*, 3757–3766.

(12) Dang, L. X. Mechanism and thermodynamics of ion selectivity in aqueous solutions of 18-crown-6 ether: a molecular dynamics study. *Journal of the American Chemical Society* **1995**, *117*, 6954–6960.

(13) Joung, I. S.; Cheatham III, T. E. Determination of alkali and halide monovalent ion parameters for use in explicitly solvated biomolecular simulations. *The journal of physical chemistry B* **2008**, *112*, 9020–9041.

(14) Zeron, I.; Abascal, J.; Vega, C. A force field of Li+, Na+, K+, Mg2+, Ca2+, Cl-, and SO 4 2- in aqueous solution based on the TIP4P/2005 water model and scaled charges for the ions. *The Journal of chemical physics* **2019**, *151*, 134504.

(15) Loche, P.; Steinbrunner, P.; Friedowitz, S.; Netz, R. R.; Bonthuis, D. J. Transferable ion force fields in water from a simultaneous optimization of ion solvation and ion–ion interaction. *The Journal of Physical Chemistry B* **2021**, *125*, 8581–8587.

(16) Chow, Y. F.; Maitland, G. C.; Trusler, J. M. Interfacial tensions of (H2O+ H2) and (H2O+ CO2+ H2) systems at temperatures of (298–448) K and pressures up to 45 MPa. *Fluid Phase Equilibria* **2018**, *475*, 37–44.

(17) Hosseini, M.; Fahimpour, J.; Ali, M.; Keshavarz, A.; Iglauer, S. H2- brine interfacial tension as a function of salinity, temperature, and pressure; implications for hydrogen geo-storage. *Journal of Petroleum Science and Engineering* **2022**, *213*, 110441.

(18) Muller, E. A.; Ervik, Å.; Mejía, A. A guide to computing interfacial properties of fluids

from molecular simulations [Article v1. 0]. *Living Journal of Computational Molecular Science* **2020**, *2*, 21385–21385.

(19) Madala, H. R. *Inductive learning algorithms for complex systems modeling*; CRC press, 2019.

(20) Ivakhnenko, A. G. Polynomial theory of complex systems. *IEEE transactions on Systems, Man, and Cybernetics* **1971**, 364–378.

(21) Ferreira, C. Gene expression programming: a new adaptive algorithm for solving problems. *arXiv preprint cs/0102027* **2001**,

(22) Jalal, F. E.; Xu, Y.; Iqbal, M.; Javed, M. F.; Jamhiri, B. Predictive modeling of swell-strength of expansive soils using artificial intelligence approaches: ANN, ANFIS and GEP. *Journal of Environmental Management* **2021**, *289*, 112420.
